# The Majority of Active *Rhodobacteraceae* in Marine Sediments Belong to Uncultured Genera: A Molecular Approach to Link Their Distribution to Environmental Conditions

**DOI:** 10.3389/fmicb.2019.00659

**Published:** 2019-04-02

**Authors:** Marion Pohlner, Leon Dlugosch, Bernd Wemheuer, Heath Mills, Bert Engelen, Brandi Kiel Reese

**Affiliations:** ^1^Paleomicrobiology Group, Institute for Chemistry and Biology of the Marine Environment, University of Oldenburg, Oldenburg, Germany; ^2^Group “Biology of Geological Processes”, Institute for Chemistry and Biology of the Marine Environment, University of Oldenburg, Oldenburg, Germany; ^3^Centre for Marine Bio-Innovation, The University of New South Wales, Sydney, NSW, Australia; ^4^Rhodium Scientific LLC, San Antonio, TX, United States; ^5^Department of Life Sciences, Texas A&M University-Corpus Christi, Corpus Christi, TX, United States

**Keywords:** benthic, diversity, pyrosequencing, phylogeny, microbial communities

## Abstract

General studies on benthic microbial communities focus on fundamental biogeochemical processes or the most abundant constituents. Thereby, minor fractions such as the *Rhodobacteraceae* are frequently neglected. Even though this family belongs to the most widely distributed bacteria in the marine environment, their proportion on benthic microbial communities is usually within or below the single digit range. Thus, knowledge on these community members is limited, even though their absolute numbers might exceed those from the pelagic zone by orders of magnitudes. To unravel the distribution and diversity of benthic, metabolically active *Rhodobacteraceae*, we have now analyzed an already existing library of bacterial 16S rRNA transcripts. The dataset originated from 154 individual sediment samples comprising seven oceanic regions and a broad variety of environmental conditions. Across all samples, a total of 0.7% of all 16S rRNA transcripts was annotated as *Rhodobacteraceae.* Among those, *Sulfitobacter*, *Paracoccus*, and *Phaeomarinomonas* were the most abundant cultured representatives, but the majority (78%) was affiliated to uncultured family members. To define them, the 45 most abundant *Rhodobacteraceae*-OTUs assigned as “uncultured” were phylogenetically assembled in new clusters. Their next relatives particularly belonged to different subgroups other than the *Roseobacter* group, reflecting a large part of the hidden diversity within the benthic *Rhodobacteraceae* with unknown functions. The general composition of active *Rhodobacteraceae* communities was found to be specific for the geographical location, exhibiting a decreasing richness with sediment depth. One-third of the *Rhodobacteraceae*-OTUs significantly responded to the prevailing redox regime, suggesting an adaption to anoxic conditions. A possible approach to predict their physiological properties is to identify the metabolic capabilities of their nearest relatives. Those need to be proven by physiological experiments, as soon an isolate is available. Because many uncultured members of these subgroups likely thrive under anoxic conditions, in future research, a molecular-guided cultivation strategy can be pursued to isolate novel *Rhodobacteraceae* from sediments.

## Introduction

The family *Rhodobacteraceae* within the *Alphaproteobacteria* is among the nine most widely distributed bacterial lineages in marine habitats (Giovannoni and Rappé, 2000; Garrity et al., 2005). They are highly abundant in the pelagic zone (Ghai et al., 2012; Haggerty and Dinsdale, 2017; Seo et al., 2017) and in algae-associated biofilms (Elifantz et al., 2013). The authors report that *Rhodobacteraceae* can comprise up to 30% of the pelagic *Alphaproteobacteria* within a coastal lagoon and up to 25% of the total bacterial community in a biofilm at the Mediterranean coast, respectively (Ghai et al., 2012; Elifantz et al., 2013). The *Rhodobacteraceae* comprise approximately 170 genera ^1^^,^^2^^,^^3^, sharing >89% identity of the 16S rRNA gene (Brinkhoff et al., 2008; Simon et al., 2017) (Supplementary Table S3). Members of this family are characterized by a high phenotypic and ecologic diversity (Pujalte et al., 2014). Their metabolism includes the utilization of various organic and inorganic compounds, sulfur oxidation, aerobic anoxygenic photosynthesis, carbon monoxide oxidation and the production of secondary metabolites (Pujalte et al., 2014). The *Rhodobacteraceae* are divided into six different phylogenetic subgroups: *Stappia*, *Amaricoccus*, *Paracoccus*, *Rhodobacter*, *Rhodovulum*, and the *Roseobacter* group (Pujalte et al., 2014).

The *Stappia* group is *sensu stricto* phylogenetically not belonging to the *Rhodobacteraceae* and is composed of aerobic or facultative anaerobic members with some species synthetizing bacteriochlorophyll *a* (Biebl et al., 2007; Pujalte et al., 2014). They exhibit various metabolic capabilities, e.g., production of bioactive compounds, carbon monoxide oxidation, aromatic ring cleavage, and degradation of oil (Buchan et al., 2001; Biebl et al., 2007; Li et al., 2011; O’Halloran et al., 2011). The three genera within the *Paracoccus* group comprise members thriving in, e.g., soils, sewage treatment plants and also hydrothermal vents (Urakami et al., 1990; Takai et al., 2009; Lee and Lee, 2013). This group is only known for a chemotrophic lifestyle, while no phototrophic activity is described (Pujalte et al., 2014). In contrast, the *Amaricoccus* group harbors photosynthetic purple non-sulfur bacteria and aerobic anoxygenic phototrophs (Suzuki et al., 1999; Boldareva et al., 2009) as well as chemoorganotrophic representatives (Maszenan et al., 1997; Lim et al., 2008). All species within the eponymous genus *Amaricoccus* were isolated from activated sludge (Maszenan et al., 1997). The *Rhodobacter* group, performing aerobic anoxygenic photoheterotrophy or the oxidation of sulfur compounds (Sorokin et al., 2005; Labrenz et al., 2009), is dominated by species isolated from freshwater and terrestrial habitats, whereas the *Rhodovulum* group mainly contains halophilic, marine species. Both subgroups comprise photosynthetic purple non-sulfur genera, which are not present in the *Roseobacter* group (Pujalte et al., 2014).

The *Roseobacter* group contains the largest proportion of described genera (72%), consisting of approximately 330 species^1^ with validly published names. The group mainly contains aerobic heterotrophs, which were identified as key players in carbon and organic sulfur cycling (González et al., 1999; Moran et al., 2003; Newton et al., 2010). In the North Sea, members of the *Roseobacter* group were found to be major players in the degradation of phytoplankton-derived organic matter (Bakenhus et al., 2017). They thrive in a great variety of marine habitats with a majority found to be particle-attached or free-living in seawater (González et al., 2000; Wagner-Döbler and Biebl, 2006; Giebel et al., 2009; Luo and Moran, 2014). In the benthic environment, the proportion of *Roseobacter* group members is often within or below the single-digit range (Buchan et al., 2005; Kanukollu et al., 2016), but in eutrophic tidal flats or brackish river sediments, they can also account for up to 10% of the bacterial communities (González et al., 1999; Lenk et al., 2012). Albeit their relative proportion seems to be small, direct quantifications of the *Roseobacter* group in North Sea sediments showed that their cell numbers can exceed those of the pelagic zone by a factor of 1000 (Lenk et al., 2012). Furthermore, the community composition of members of the *Roseobacter* group in benthic and pelagic systems differs significantly, showing an increase in diversity from surface waters to the seafloor with specific compositions in the free-living and attached fractions (Stevens et al., 2005; Kanukollu et al., 2016). While the distribution of the *Roseobacter* group in sediments is increasingly investigated (e.g., Lenk et al., 2012; Kanukollu et al., 2016; Pohlner et al., 2017), there is a paucity of information on benthic *Rhodobacteraceae*.

*Rhodobacteraceae* are frequently neglected in studies on benthic microbial diversity, as these investigations often focus on the most abundant community members or those driving characteristic biogeochemical processes (e.g., Orphan et al., 2001; Llobet-Brossa et al., 2002; Mills et al., 2008; Graue et al., 2012). A major problem in resolving the diversity of *Rhodobacteraceae* is the high proportion of sequences affiliated to uncultured members of this family. Buchan et al. (2005) reported that it is not possible to access relevant physiological information for two-thirds of the so-far identified diversity of the *Roseobacter* group members through studies of cultured organisms from marine habitats. Meanwhile, the number of isolates increased, but many sequences could still not be assigned to cultured genera. Although they are annotated as *Rhodobacteraceae*, they remain assigned as “uncultured” on genus level. In sediments along a Pacific transect, we found that 84% of all *Rhodobacteraceae*-assigned operational taxonomic units (OTUs) could not be affiliated to cultured relatives (Pohlner et al., 2017). This problem even increases from the sea surface down to the seafloor and deeper into the sediments (Kanukollu et al., 2016). Although 25% of all *Rhodobacteraceae* isolates (known in 2014) are of benthic origin (Pujalte et al., 2014), there is an unexplored diversity that hampers the interpretation concerning their role in nutrient cycling.

A first step to address this issue is to correlate the distribution of the uncultured representatives to a wide range of environmental conditions. In the present study, we conducted a global survey on the distribution and diversity of active members of benthic *Rhodobacteraceae*. The sediments were collected at seven sampling sites exhibiting different biogeochemical settings and sediment depths from the surface to several meters below seafloor (mbsf). To identify the metabolically active *Rhodobacteraceae*, 154 individual samples were subjected to RNA extraction and pyrosequencing of 16S rRNA transcripts. The overall community structures were previously described in several publications (Mills et al., 2012; Reese et al., 2013, 2014, 2018). Because these datasets were processed over several years, all sequences first had to run through the same bioinformatic pipeline with a unified quality control and phylogenetic reclassification. All sequences belonging to the *Rhodobacteraceae* were used to correlate their community composition and distribution pattern to the different environmental conditions. Additionally, phylogenetic trees were constructed using various algorithms to identify the branching of OTUs assigned to so-far uncultured members of this family.

We hypothesize that the overall composition of benthic *Rhodobacteraceae* and the presence of representative members is driven by the environmental conditions within the sediments. Thus, individual members of this family might specifically inhabit different oceanic regions and sediment layers. Overall, the current study reveals a deeper insight into the phylogeny, diversity, and distribution of active members of the *Rhodobacteraceae* in sediments globally.

## Materials and Methods

### Origin and Sampling of Sediments

**FIGURE 1:**
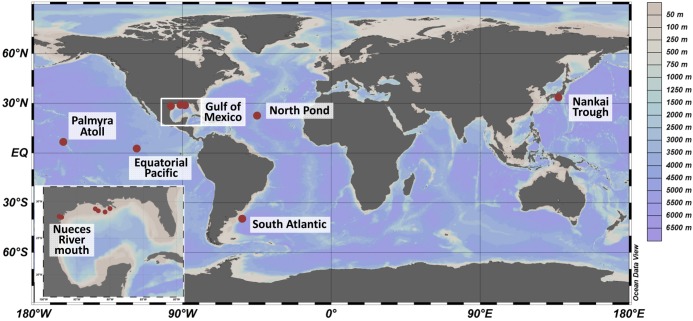
Sampling sites worldwide (large panel) and in the Gulf of Mexico (small panel). Sediments were collected from 2007 to 2012 during several expeditions. The color scale represents water depths at the various sites. The map was created using OceanDataView (Schlitzer, 2018).

Sediment samples were collected at seven locations worldwide (Figure 1 and Supplementary Table S1) during various oceanographic and field expeditions between 2007 and 2012. The shallowest sites with a water depth of 3 mbsl were located in the mouth of Nueces River, near Corpus Christi Bay (Corpus Christi, TX, United States) and were sampled in 2009 as described by Reese et al. (2014). Several locations within the Gulf of Mexico were sampled between 2008 and 2012 during expeditions Mechanisms Controlling Hypoxia (MCH) 11, 12, 14, 19 and 21 on board the R/V Pelican and sediments were treated as reported by Reese et al. (2013). On the Palmyra Atoll, surface sediments were collected at 50 m water depth (unpublished). The sampling sites located in the Nankai Trough, South Atlantic, Equatorial Pacific, and western flank of the Mid-Atlantic Ridge (herein referred to as North Pond) exhibited water depths of several thousand meters (3000–4500 mbsl). Sampling sites in the Nankai Trough, were investigated in 2007 during Integrated Ocean Drilling Program (IODP) Expedition 316: NanTroSEIZE Shallow Megasplay and Frontal Thrusts (Mills et al., 2012). The Equatorial Pacific samples were collected during IODP Expedition 327: Pacific Equatorial Age Transect in 2009 (unpublished). Sediments from North Pond were collected down to 74 mbsf during IODP Expedition 336: Mid-Atlantic Ridge Microbiology in 2011 (Reese et al., 2018). Prior to all IODP drilling activities, microspheres were added to the drill mud to track intrusion of the fluid into the sediment core. Cores designated for microbiological and geochemical analyses were sectioned using aseptic techniques into 10 cm whole round core segments. The sub-sectioned cores were frozen immediately on board at -80°C. Samples identified as South Atlantic were collected using gravity coring from the Argentine Basin during R/V Meteor expedition M78/3 (unpublished). Details on geochemical analyses and sediment characteristics were described in the individual publications and were summarized in Supplementary Table S1.

### Extraction and Molecular Analysis

As the focus of the individual sampling campaigns was the investigation of metabolically active community members, ribosomal RNA was analyzed, exclusively. Total RNA was extracted from frozen sediment using a phenol–chloroform method modified from previously published methods (Reese et al., 2013, 2018). In brief, an aliquot of sediment was chipped from the center of frozen sediment cores using an aseptic technique, which included flame sterilized utensils, donning facemask and hairnet, and performing work in a UV-sterilized and HEPA-filtered biosafety hood. A negative blank containing no sample (i.e., extraction control) was extracted alongside each set of sample extractions to verify lack of contamination. Details about the molecular analysis are published elsewhere and a brief overview is provided herein. Extracts were checked for quality on a NanoDrop spectrophotometer (Thermo Fisher; Wilmington, DE, United States) and quantified on a Qubit fluorometer (Thermo Fisher; Wilmington, DE, United States). Residual DNA was removed using a DNAse enzyme (Ambion Turbo DNA Free; Austin, TX, United States) following the manufacturer protocol. RNA was reverse-transcribed to complementary DNA (cDNA) using moloney murine leukemia virus (MMLV) reverse transcriptase (Promega; Madison, WI, United States) and the *Bacteria*-specific 16S rRNA gene primer, 518R (5′-CGT ATT ACC GCG GCT GCT GG-3′; Nogales et al., 1999). Resulting cDNA was PCR-amplified using *Bacteria*-specific 16S rRNA gene forward primer, 27F (5′-AGR GTT TGA TCM TGG CTC AG-3′; Giovannoni et al., 1991) and reverse primer 518R. Reaction conditions were 95°C for 5 min, 35 cycles of 95°C for 30 s, 50°C for 30 s, and 72°C for 30 s, and 72°C for 10 min. The raw RNA extract was PCR-amplified following the same protocol to verify lack of contaminating DNA.

### Pyrosequencing

Reverse transcribed RNA (cDNA) were sequenced using a 454 FLX Sequencer (454 Life Science; Branford, CT, United States) at the Research and Testing Laboratory (Lubbock, TX, United States). Primers 28F (5′-GAG TTT GAT CNT GGC TCA G-3′) and 519R (5′-GTN TTA CNG CGG CKG CTG-3′) were used, which spanned the hypervariable V1–V3 region of the 16S rRNA gene. The extraction blanks were PCR-amplified at 55 cycles to identify potential contamination and sequenced despite lack of visible amplification. Sequences were screened for minimum read length of 250 bp and minimum quality scores of 25, and further screened as described below. The 16S rRNA transcript nucleotide sequences have been deposited in the National Institutes of Health (NIH) GenBank database under BioProject numbers PRJNA527322 (Nueces River mouth), PRJNA526779 (Gulf of Mexico), PRJNA527340 (South Atlantic), PRJNA527344 (Equatorial Pacific), PRJNA308331 (North Pond), and Sequence Read Archive numbers SRP010369 (Gulf of Mexico) and SRA049352 (Nankai Trough).

### Processing and Analysis of 454-Data

As the existing dataset was compiled from individual studies focusing on metabolically active microbial communities, only 16S rRNA transcripts, but no 16S rRNA gene sequences were available for our analysis. Due to the fact that the sediments were collected over several years, the generated datasets needed to be unified. Therefore, all raw data were processed together in a bioinformatic pipeline containing quality control and phylogenetic classification to generate OTUs.

Datasets of 16S rRNA transcript sequences were demultiplexed and quality filtered with QIIME version 1.8 (Caporaso et al., 2010) using the *split_libraries.py* script. Filtering included the removal of sequences shorter than 200 bp, with homopolymers longer than 8 bp, more than three mismatches in the forward primer, and more than three ambiguous bases. Sequences of all samples were subsequently concatenated to one file. Cutadapt version 1.16 was used to truncate remaining reverse primer sequences (Martin, 2011) prior to denoising with Acacia version 1.53b (Bragg et al., 2012). Sequences were sorted by decreasing length and clustered at 3% dissimilarity (Wemheuer et al., 2015) using the ulcust_fast algorithm implemented in USEARCH (version 8.1.1861; Edgar, 2010). Singletons (sequences appearing in only one sample with one sequence) were removed according to Schneider et al. (2013). Chimeric sequences were removed using UCHIME (Edgar et al., 2011) in *de novo* mode and subsequently in *reference* mode using the SILVA SSURef 128 NR database (Quast et al., 2013) as reference implemented in USEARCH (Edgar, 2010). To determine taxonomy, one sequence representing each OTU was classified by BLAST against the Silva SSURef 128 NR database (Camacho et al., 2009).

### Phylogenetic Analysis

Operational taxonomic units were generated using a threshold of 97% sequence identity to obtain maximum phylogenetic resolution and information on uncultured members of the *Rhodobacteraceae*. Because a total of 198 OTUs could not be assigned to cultured genera, we focused on the 45 most abundant *Rhodobacteraceae*-OTUs assigned as “uncultured” for phylogenetic analysis. The other 153 OTUs (each contributing less than 0.2% to the uncultured fraction) were regarded as “low abundant” to keep the trees clear and readable. Sequences of the first described type strain for every genus within the *Rhodobacteraceae* were extracted from the SILVA database (SSU Ref NR99 132). The present phylogeny of the family *Rhodobacteraceae* was used as described by Pujalte et al. (2014) and extended by newly cultured genera listed as prokaryotic names with standing in nomenclature ^4^, in the NCBI taxonomy browser ^5^ and the Global Biodiversity Information Facility ^6^. For the assignment of all genera into the six different subgroups within the *Rhodobacteraceae*, see Supplementary Table S3. To display the next relatives of the OTUs identified as “uncultured,” the consensus sequences of these OTUs (approximately 400 bp length) were aligned using SILVA SSU Ref NR99 132 (Pruesse et al., 2012) and after a manual check of the alignment, phylogenetic trees were calculated. First, the sequences of the 45 OTUs assigned as “uncultured” were added to backbone tree of the SILVA database (SSURef NR99 132) by the maximum-parsimony method using the Quick-Add function in ARB (version 6.0.2; Ludwig et al., 2004). Furthermore, the 45 predominant sequences assigned to uncultured members, the type strain of every genus within the *Rhodobacteraceae* as well as 50 sequences of *Rhizobium* sp. (also members of the *Alphaproteobacteria*) as root were used to calculate different trees using maximum parsimony (once), maximum likelihood and neighbor joining, each in triplicates. Afterward, the branching of the OTU sequences with the type strains was compared between the individual trees.

### Statistical Data Analysis

Statistical analyses were performed in R (version 3.5.2; R Core Team, 2018). Following packages were used: vegan (version 2.5-2; Oksanen et al., 2018), permute (version 0.9-4; Simpson, 2016), pvclust (version 2.0-0; Suzuki and Shimodaira, 2015), and gclus (version 1.3.1; Hurley, 2012). To reduce the complexity of the dataset in further analyses, OTUs affiliated to the same cultured genus were combined. Due to largely inconsistent sampling depth between sites, samples of the same site were pooled in several depth categories: the upper 20 cm were divided into 2 cm increments, the other depth categories were defined as 0.2–10 mbsf, 10–20 mbsf, and 20–100 mbsf (see Supplementary Table S1 for original sediment depths). As a total of 198 *Rhodobacteraceae*-affiliated OTUs could not be affiliated to previously described genera, we focused on the 45 most abundant *Rhodobacteraceae*-OTUs assigned as “uncultured” analyzing the richness of the communities. Using all detected OTUs, Bray–Curtis distances were calculated between sampling sites and associated depth categories and non-metric multidimensional scaling (NMDS, *k* = 2, 10,000 permutations) was performed to visualize the data. Spearman’s rank correlations were calculated between all bacterial OTU abundances and the environmental parameters. *P*-values were adjusted for multiple testing according to Benjamini and Hochberg (1995). Correlations with *p* ≤ 0.05 were considered significant. Final image editing was done in inkscape^7^ (version 0.92).

## Results

### On Average, 0.7% of the 16S rRNA Transcripts Were Assigned to the *Rhodobacteraceae*

**FIGURE 2:**
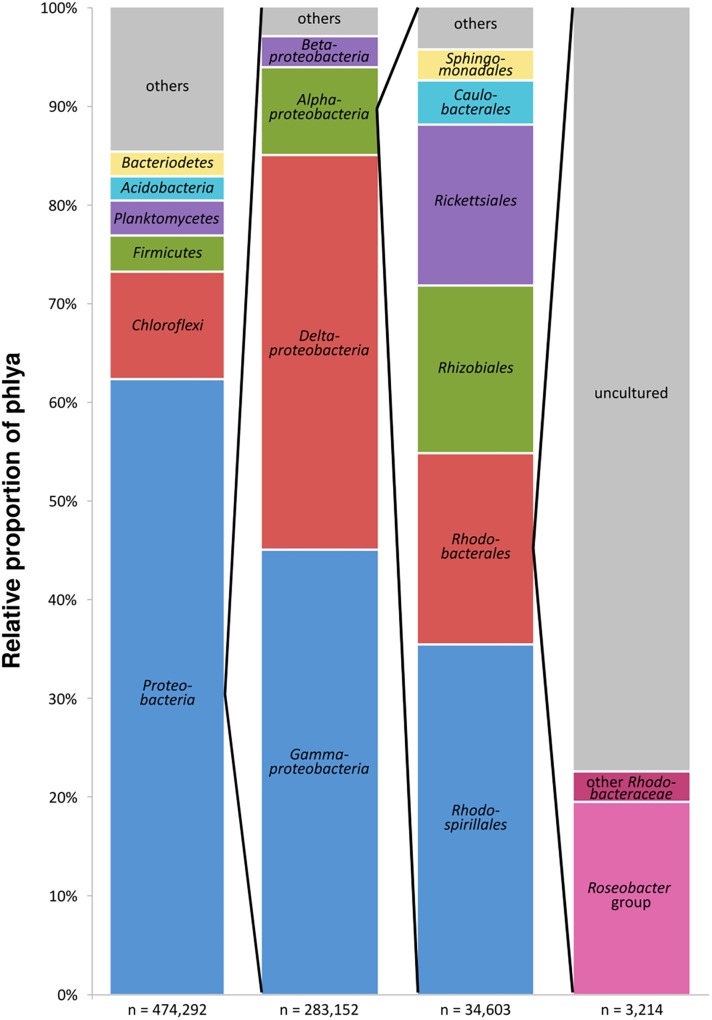
Composition of the benthic, metabolically active microbial community based on bacterial 16S rRNA transcripts in the entire dataset displayed in the different phylogenetic levels (*n* = sum of sequences in the respective phylogenetic level, “others” includes all phyla < 2%).

The entire dataset of all bacterial 16S rRNA transcripts from all seven sampling sites (154 samples) consisted of approximately 900,000 sequences. After removal of low-quality and non-bacterial reads, overall 474,290 high-quality sequences were obtained. Those were grouped into 37,747 OTUs (97% sequence identity level). A total of 62.3% of the OTUs were classified as *Proteobacteria*, followed by *Chloroflexi* (10.8%), *Firmicutes* and *Planctomycetes* (3.7% and 3.6%, respectively), *Bacteroidetes* and *Acidobacteria* (both 2.5%) and several others in minor proportions (Figure 2). Within the *Proteobacteria*, 45.0% of the OTUs could be assigned to *Gammaproteobacteria*, 39.9% to *Deltaproteobacteria*, 8.9% to *Alphaproteobacteria*, and 3.1% to *Betaproteobacteria*. The sequences assigned to *Alphaproteobacteria*, in turn, consisted of the orders *Rhodospirillales* (35.4%), *Rhodobacterales* (19.3%) with the *Rhodobacteraceae* as the only family within this order, *Rhizobiales* (17.0%), and *Rickettsiales* (16.3%). The 16S rRNA transcripts assigned to the *Rhodobacteraceae* family (3,214 sequences) were grouped into 243 OTUs. A total of 19.5% of these OTUs were assigned to cultured genera within the *Roseobacter* group, whereas only 3.1% were related to cultured members of the other subgroups within the *Rhodobacteraceae.* The majority of the OTUs (77.5% of the *Rhodobacteraceae* community) were assigned to uncultured representatives (Figure 2). Within the entire dataset of all investigated sediments, the *Rhodobacteraceae* accounted for 0.7% of the active bacterial communities. Of these, 0.5% were assigned to uncultured and 0.2% to cultured members of the *Rhodobacteraceae* with *Sulfitobacter*, *Paracoccus* and *Phaeomarinomonas* in highest abundances (for relative abundances of individual OTUs at the various sampling sites see Supplementary Table S2).

### The Majority of *Rhodobacteraceae*-Affiliated OTUs Assigned as “Uncultured” Fall Into Defined Phylogenetic Clusters

**FIGURE 3:**
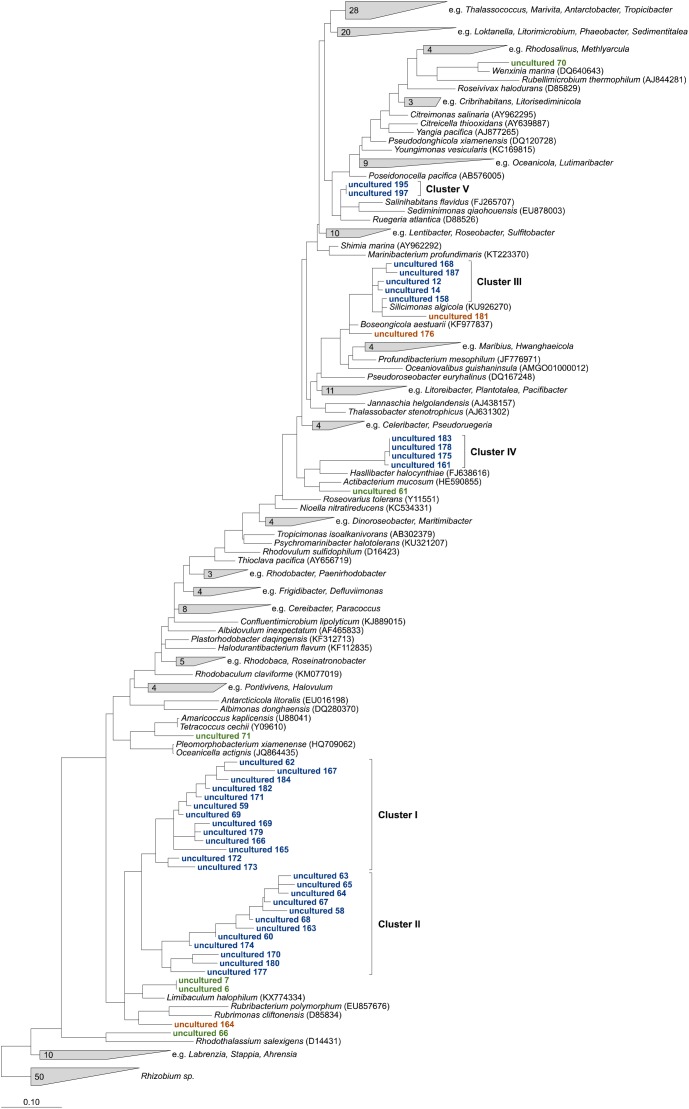
Maximum likelihood tree highlighting the position of consensus sequences of the 45 most abundant *Rhodobacteraceae*-OTUs assigned as “uncultured” relative to other members of the family. The tree was created using ARB (Ludwig et al., 2004) and rooted by sequences of the genus *Rhizobium*. To keep the tree clear and readable, type strains that were not related to the “uncultured” sequences were collapsed into single groups. Sequences in green could clearly be related to next relatives, orange ones changed positions in comparison to neighbor joining and maximum parsimony trees. Blue sequences always formed consistent clusters.

Overall, 198 of 243 OTUs could not be affiliated to any cultured relatives within the family and were assigned as “uncultured.” The majority of 153 OTUs were “low abundant” and contributed each on average less than 0.2% to the uncultured fraction, while 75% of the uncultured *Rhodobacteraceae* were represented by the remaining 45 OTUs. The phylogenetic neighborhood of those was identified by constructing phylogenetic trees using their consensus sequences. To obtain the most robust phylogenetic affiliation of the sequences (read length up to 451 bp), multiple phylogenetic algorithms including maximum parsimony, maximum likelihood, and neighbor joining were tested. After comparing the architecture of the individual trees, the maximum likelihood tree was chosen to be the most accurate and reproducible (Figure 3).

Six OTU sequences clearly branched with the same cultured relatives in all trees tested and could be related to those genera (Figure 3, marked in green). Thus, the sequences of OTUs identified as “uncultured 6” and “uncultured 7” were consistently related to the type strain of the genus *Limibaculum*, and OTUs identified as “uncultured 61,” “uncultured 66,” “uncultured 70” and “uncultured 71” branched with the type strains of *Actibacterium*, *Rhodothalassium*, *Wenxinia*, and *Tetracoccus/Amaricoccus*, respectively. Some of the OTUs assigned as “uncultured” could not be assigned to any groups because their position was inconsistent within the individual trees (Figure 3, orange). However, some of the sequences grouped together and formed clusters despite the algorithm used (Figure 3, blue). Two large clusters (I and II) consisting of 13 and 12 sequences, respectively, clustered with each other in all trees. Cluster III always branched closely to the type strain of *Silicimonas*, but could not clearly be related to this genus. Blasting of the individual sequences against the NCBI database revealed identities of 95% (“uncultured 187”)–99% (“uncultured 158”) to *Silicimonas algicola*. The two smaller clusters IV and V (comprising 4 and 2 sequences, respectively) were related to *Actibacterium* and *Pseudophaeobacter* in the neighbor joining trees, but the nearest relatives were different in the maximum likelihood trees.

### The Geographical Location Influences the *Rhodobacteraceae* Community Composition

**FIGURE 4:**
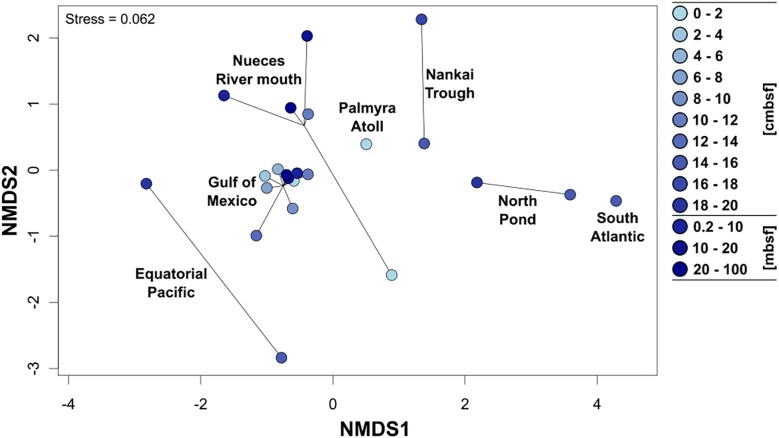
Non-metric multidimensional scaling plot (NMDS based on Bray–Curtis distances) of the active *Rhodobacteraceae* community compositions based on the different sampling sites. Cycles show the community compositions at the specific site and the color of the cycles indicates increasing sediment depths (light blue: surface, dark blue: down to 100 m below seafloor).

To allow a comparison of the community composition of metabolically active *Rhodobacteraceae* among the different sampling sites, all 154 individual samples were grouped in several depths categories: the upper 20 cm were divided into 2 cm increments, the other depth categories were defined as 0.2–10 mbsf, 10–20 mbsf, and 20–100 mbsf. In general, all surface near samples from the Gulf of Mexico, the Nueces River mouth, and the Palmyra Atoll revealed a similar composition of *Rhodobacteraceae* communities as indicated by NMDS analysis (Figure 4). This was especially pronounced for the Gulf of Mexico, which was analyzed in highest resolution. The various depths of the Nueces River mouth sediments also formed a consistent cluster, except the very top layer (0–2 cmbsf). Subsurface sediments of the Nankai Trough, North Pond, the South Atlantic, and the Equatorial Pacific (all 0.2 mbsf and deeper) exhibited individual clusters, each clearly separated from other locations. Here, the active communities of the two Atlantic Ocean sites (South Atlantic and North Pond) and the Nankai Trough were more similar to each other than to those from the Equatorial Pacific.

### The Top Layer of Surface Sediments Revealed a Greater *Rhodobacteraceae* Richness Than the Subsurface

**FIGURE 5:**
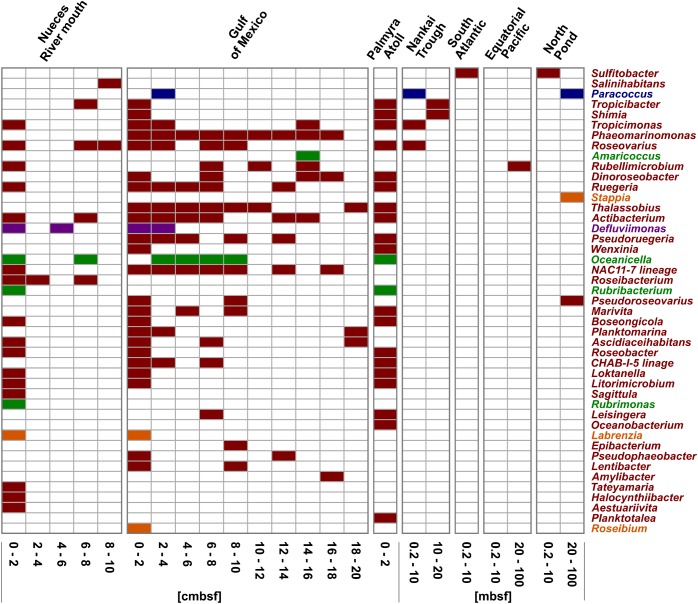
Distribution of the OTUs affiliated to cultured representatives within the *Rhodobacteraceae*. OTUs are sorted by their relative abundance on the total bacterial community. Sample locations are arranged by water depth and then by sediment depth. The affiliation of OTUs to the phylogenetic subgroups is displayed by red = *Roseobacter*, orange = *Stappia*, green = *Amaricoccus*, purple = *Rhodobacter*, and blue = *Paracoccus*.

In general, every sampling site and each depth layer was characterized by individual community patterns of active *Rhodobacteraceae* (Figure 5). The samples of the very top layer of the Nueces River mouth, the Gulf of Mexico and the Palmyra Atoll showed the highest species richness as indicated by a high number of different OTUs. In all samples from 0–2 cmbsf, OTUs affiliated to *Tropicimonas*, *Roseovarius*, *Ruegeria*, *Actibacterium*, *Boseongicola*, *Roseobacter*, *Loktanella*, and *Litorimicrobium* were detected. However, the subsurface and especially the deep subsurface were generally characterized by low species richness. In the Gulf of Mexico, some OTUs of the very top layer were also present in the different subsurface layers down to 20 cmbsf (e.g., *Thalassobius*, *Phaeomarinomonas*, *Actibacterium*, NAC11-7 lineage). Those resemble *Rhodobacteraceae* communities from the seafloor prevailing in deeper sediments in minor proportions. Within the deep subsurface (>10 mbsf) of the Nankai Trough, the Equatorial Pacific, and the North Pond *Rhodobacteraceae*-affiliated OTUs (namely *Tropicibacter*, *Shimia*, *Rubellimicrobium*, *Pseudoroseovarius*, *Paracoccus*, and *Stappia*) were only detected sporadically. Some OTUs only occurred at single sites.

**FIGURE 6:**
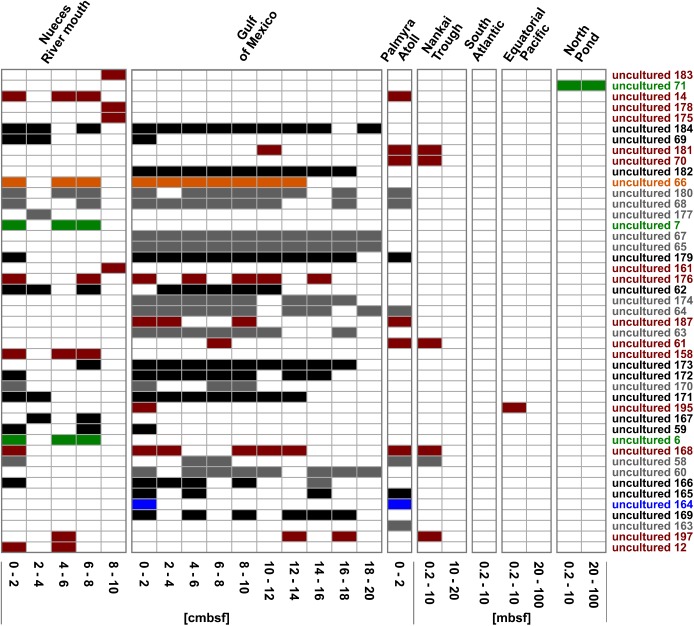
Distribution of the OTUs affiliated to uncultured representatives within the *Rhodobacteraceae*. OTUs are sorted by their relative abundance on the total bacterial community. Sample locations are arranged by water depth and then by sediment depth. The affiliation of OTUs to the phylogenetic subgroups is displayed by red = *Roseobacter*, orange = *Stappia*, green = *Amaricoccus*, black = cluster I, gray = cluster II, and blue = unclear assignment.

Focusing on the community composition formed by the 45 most abundant *Rhodobacteraceae*-OTUs affiliated to active, but so-far uncultured members of this family, the distribution was again specific for every site and depth (Figure 6). Overall, around 42% of these OTUs were exclusively found in individual sampling sites. In general, a decreasing richness with increasing sediment depth was also found in the uncultured fraction. A main difference was the continuous distribution of several OTUs over the upper 20 cm in the Gulf of Mexico samples. Those mostly belonged to clusters I and II (black and gray bars in Figure 6). At some subsurface sites (South Atlantic: >0.2 mbsf, Nankai Trough: >10 mbsf, and Equatorial Pacific: >20 mbsf) only a few OTUs affiliated to cultured representatives or the 153 “low abundant” OTUs were detected.

### One-Third of All Detected *Rhodobacteraceae-*OTUs Correlate Significantly to the Sedimentary Settings

**FIGURE 7:**
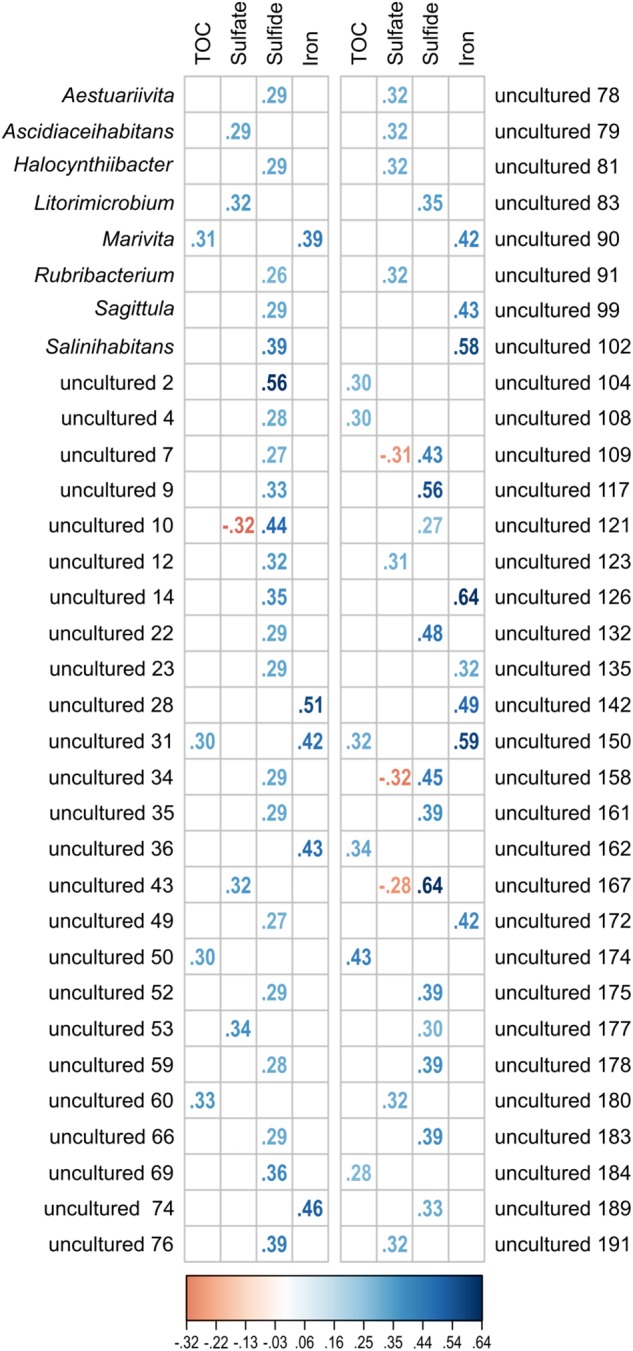
Significant correlation (*p* ≤ 0.05) of single OTUs to the environmental parameters TOC, sulfate, sulfide, and iron (Fe^2+^). All OTUs related to cultured and uncultured *Rhodobacteraceae* were included in the analysis. Displayed are the Spearman’s rank correlation coefficients (*r*_s_). While increasing blue colors show positive correlations, red colors indicate negative values.

The investigated dataset of 16S rRNA transcripts was accompanied by environmental metadata such the geographical setting (location, water and sediment depth) and geochemical parameters (Supplementary Table S1). Due to altering priorities during the various sampling campaigns, some geochemical analyses were not performed for all 154 samples (TOC: *n* = 114; porewater sulfate: *n* = 113, sulfide: *n* = 105 and Fe^2+^: *n* = 83). While the geographical setting showed no significant correlation, approximately one-third of all detected *Rhodobacteraceae*-affiliated OTUs were significantly correlated to the geochemical parameters (*p* ≤ 0.05). Those OTUs (58 assigned as “uncultured” and 8 related to cultured genera) were categorized regarding their Spearman’s rank correlation coefficients (*r*_s_) (Figure 7). Overall, 59 OTUs showed one positive correlation to a single environmental parameter, only. This was especially the case for porewater sulfide concentrations (35 OTUs). Spearman’s rank coefficients were low for most correlations (*r*_s_ < 0.5), but highest with respect to sulfide concentrations (*r*_s_ > 0.5 for OTUs “uncultured 2,” “uncultured 117,” and “uncultured 167”). Some OTUs were correlated to both, sulfate (*r*_s_ ≈-0.3) and sulfide (*r*_s_ ≈ +0.5). Another combination was a positive correlation to TOC (*r*_s_ ≈ 0.3) and iron concentrations (*r*_s_ = 0.4–0.6).

## Discussion

### Revisiting Already Existing Datasets Offers the Opportunity to Answer Novel Ecological Questions

Over the last years, pyro- and Illumina sequencing of 16S rRNA genes and transcripts were used in many studies to investigate the microbial diversity of marine sediments (e.g., Zhu et al., 2013; Lee et al., 2014; Lentini et al., 2014; Mahmoudi et al., 2015). The vast amount of generated data was mostly processed to display the overall community composition at different locations. However, the technique offers a much deeper insight into different phylogenetic levels to answer more sophisticated questions, e.g., on the distribution of specific phyla with low abundance. The compiled dataset presented here, was previously published in individual studies to describe the active community compositions at the particular locations (Mills et al., 2012; Reese et al., 2013, 2014, 2018). The whole process of reanalyzing the data was only possible due to an intense collaboration between all coauthors of this study and the willingness to provide the raw data. Thus, sharing and revisiting already existing sequencing data can help to investigate global distribution patterns without redundant sample acquisitions.

Overall, the challenge was the unification of the single datasets that were collected over several years to define OTUs and to reclassify their affiliation according to the newest taxonomic database. The crucial point was the reprocessing of raw data by a unified bioinformatic pipeline, including filtering by quality control and removal of singletons. As our phylum of interest was the family *Rhodobacteraceae*, we have extracted the respective OTUs out of all bacterial 16S rRNA transcripts to unravel their global distribution within sediments. Focusing on this specific family resulted in a relatively low number of OTUs per sample (on average 21 reads). We addressed this issue by pooling the 154 individual samples. Thus, the dataset was separated into the described depths categories which resulted on average in 153 reads for the single depth categories at each sampling site. This number is still low, but allows a more sound statistical analysis and reliable interpretation.

### Choosing the Appropriate Reference Database for Taxonomic Classifications Is Crucial for Analyzing Lower Phylogenetic Levels

As next-generation sequencing generates huge amounts of data that need to be archived, shared, and analyzed, the database used for taxonomic affiliation is essential in handling high-throughput data (Metzker, 2010; Zhulin, 2015). Although similar information is deposited in these databases, they were developed for different research questions and curated by various organizations leading to structural heterogeneities (Zhulin, 2015). The online database collection of “Nucleic Acids Research” currently comprises >1700 individual databases^8^ with annually increasing numbers (Rigden et al., 2016; Galperin et al., 2017). Key reference databases for studies on microbial diversity are the SILVA, the RDP (Ribosomal Database Project) and the Greengenes database (McDonald et al., 2012; Quast et al., 2013; Cole et al., 2014; Zhulin, 2015). Although all of them provide a collection of 16S rRNA gene sequences for comparisons, differences in their size and resolution lead to discrepancies in the taxonomic classifications of a given sequence. Using the SILVA and RDP databases, sequences can be classified down to the genus level, while Greengenes allows classification down to the species level, but displays the smallest number of nodes compared to both other databases (Balvočiūtė and Huson, 2017). A comparison of the commonly used reference databases revealed that around 70% of all phyla and classes, 63% of all orders and approximately 90% of all families and genera are unique to either SILVA, RDP, Greengenes or the NCBI database (Balvočiūtė and Huson, 2017). This may lead to erroneous affiliations that are not comparable across all databases. While the taxonomy annotation error rates of SILVA, RDP and Greengenes were estimated to be only 0.2–2.5% by Kozlov et al. (2016), it was recently shown that the error rate was approximately 10% for the RDP database and around 17% for Greengenes and SILVA, respectively (Edgar, 2018).

As the focus of our study was set on the *Rhodobacteraceae*, we compared the SILVA database (SSUref128) with the taxonomies of the Greengenes and RDP databases^9^. While the *Rhodobacteraceae* are subdivided into 133 genera within the SILVA taxonomy, the resolution of Greengenes and RDP only includes 23 and 93 genera of this family, respectively. During our studies, all databases were frequently updated. Mapping the taxonomies of the different databases on the newest SILVA release (SSUref132) revealed an increase of 30 genera for RDP, 6 genera for SILVA, and no change in Greengenes. This example shows how differently databases respond to quick changing developments in phylogeny and taxonomy. The choice of the database can limit the phylogenetic resolution of the results, and the outcome is strongly dependent on single genera being present or absent in the respective databases (Beiko, 2015). Although, we determined the SILVA database to be most appropriate at the time of this publication for investigating the abundance and distribution of the *Rhodobacteraceae* in marine sediments, 77.5% of the *Rhodobacteraceae*-affiliated OTUs could not be related to cultured representatives (3% dissimilarity) and remained assigned as “uncultured.”

### OTUs Assigned as “Uncultured” Are Preferably Spread Among Subgroups Within the *Rhodobacteraceae* Other Than the *Roseobacter* Group

As investigations of microbial communities often use 16S rRNA gene sequences and thus, focus on the present communities only, the active fraction of the microbial communities is largely uncharacterized. Therefore, in this study 16S rRNA transcripts were used to gain deeper insights into the metabolically active community members, although this approach might not describe the entire *Rhodobacteraceae* communities. The OTUs related to cultured *Rhodobacteraceae* are mainly affiliated to the *Roseobacter* group (80%) and in minor proportions to the *Rhodobacter*, *Rhodovulum*, *Amaricoccus*, *Paracoccus*, and *Stappia* group. Thus, the OTUs related to the *Roseobacter* group are slightly overrepresented, as this group contains 72% ^10^ of all cultured genera within the *Rhodobacteraceae* (Pujalte et al., 2014). In contrast, among the 45 most abundant OTUs assigned to uncultured representatives, only one-third was related to the *Roseobacter* group. In the phylogenetic analysis, the clusters I and II distantly branched with members of the *Amaricoccus* group. Thus, a large part of the diversity of so-far uncultured *Rhodobacteraceae* is hidden in the subgroups other than the *Roseobacter* group. Those subgroups should be a primary target in future investigations to unravel their physiological properties. However, the generally high proportion of uncultured microorganisms hampers the interpretation concerning their role in global nutrient cycling. This is especially pronounced in benthic environments as we have exemplarily shown in this study and previous *Rhodobacteraceae*-related investigations (Kanukollu et al., 2016; Pohlner et al., 2017). Targeting uncultured family members to specifically investigate their capabilities through physiological experiments and genomic analyses would fill in the knowledge gap that currently exists (Giovannoni and Stingl, 2007).

### Members of the *Rhodobacteraceae* Are Widespread Within Different Marine Sediments

In this study, the focus was on the active fraction of *Rhodobacteraceae* within coastal and open ocean sediments which were characterized by different biogeochemical settings and redox conditions. The dataset included surface as well as deeper sediments collected at seven different sampling sites. It could be shown that in all these sediments, metabolically active members of the *Rhodobacteraceae* can be found, even if their abundance might be low. Looking at the entire (active and present) *Rhodobacteraceae* communities, their occurrence was previously described for a broad variety of sedimentary settings. Thus, representatives of the family were found in mangrove sediments in Brazil and brackish estuaries in the southeastern United States (Marcial Gomes et al., 2008; Andreote et al., 2012). Furthermore, *Rhodobacteraceae* are especially present in coastal sediments such as the German Wadden Sea, coastal sediments from the Sea of Okhotsk and in microbial mats of a Dutch barrier island (González et al., 1999; Inagaki et al., 2003; Bolhuis and Stal, 2011; Lenk et al., 2012). Todorova et al. (2014) also reported the presence of members of this family in coastal sediments of the Black Sea and Diaz et al. (2013) found them in sediments from the Bahamas Archipelago. In sediments of the Nankai Trough the active fraction of *Rhodobacteraceae* of the presented dataset consisted of uncultured representatives and members of the *Paracoccus* and *Roseobacter* group. In another study on cold seep sediments at this location, *Rhodobacter*-related sequences were reported (Li et al., 1999). Additionally, *Rhodobacteraceae* are also present at high active, hydrothermal sites, e.g., a saline mud volcano in Italy and sediments of a hydrothermal vent in the Mid-Atlantic Ridge (Yakimov et al., 2002; López-Garcia et al., 2003). Even in polar regions, benthic *Rhodobacteraceae* contribute the microbial communities as described for Antarctic continental shelf sediments and deep-sea sediments along the Antarctic Polar Front and the Pacific Arctic Ocean (Bowman and McCuaig, 2003; Powell et al., 2003 Li et al., 2009). However, the general composition of benthic *Rhodobacteraceae* communities is always specific for each geographical location. Overall, members of this family are present in all kinds of marine sediments, but seem to be more abundant in coastal sites with high nutrient availability.

### Physiological Properties of the *Rhodobacteraceae* Represented by OTUs Assigned as “Uncultured” Can Be Predicted by Identifying Their Phylogenetic Relationship

A comparison of phylogenetic trees calculated with different algorithms can help to get a robust affiliation as shown for some of the OTUs detected in this study. For instance, OTUs “uncultured 61” and “uncultured 70” were clearly related to *Actibacterium* and *Wenxinia*, respectively. All members of the genus *Actibacterium* were isolated from the marine environment and putatively depend on salt for growth (Lucena et al., 2012; Li et al., 2014; Park et al., 2014b; Lin et al., 2016; Guo et al., 2017). The type strain of this genus, *A. mucosum*, requires a complex ionic composition and does not grow on media with only NaCl or KCl added (Lucena et al., 2012; Park et al., 2014b). All isolates of the genus *Wenxinia* have a benthic origin and were described to be aerobic, heterotrophic using different sugars for growth (Ying et al., 2007; Park et al., 2014a). For both genera, the ability to reduce nitrate to nitrite has been described previously (e.g., *A. atlanticum*; Li et al., 2014; *W. marina*, Ying et al., 2007), explaining the presence of the OTU related to this genera in Nankai Trough sediments at a depth of 0.2–10 mbsf. Other OTUs are related to *Limibaculum* (“uncultured 6” and “uncultured 7”) and *Rhodothalassium* (“uncultured 66”) and were detected in surface sediments of the Nueces River mouth and the Gulf of Mexico. *L. halophilum* has been shown to be aerobic, hydrolyzing gelatin, using some sugars and also more complex substrates, e.g., potassium 5-ketogluconate (Shin et al., 2017). The isolate of the genus *Rhodothalassium* has grown preferably photoorganotrophically under anoxic conditions in the light, but can also thrive under microoxic to oxic conditions in the dark (Drews, 1981; Imhoff et al., 1998).

However, next generation sequencing of 16S rRNA genes and transcripts is still limited to a few hundred base pairs, resulting in partial sequences, only (Metzker, 2010). The consequence of using short reads is a clear phylogenetic classification on the family level, but in many cases no exact assignment to a specific genus. In our investigation, the sequences of some OTUs changed their position depending on the algorithm used for phylogenetic analyses. A clear physiological classification of the organisms behind the sequences can only be achieved by classical isolation. Until these isolates are available, we have chosen the approach to classify the “uncultured” by integrating their 16S rRNA gene sequences into the phylogenetic tree to infer their physiological properties from their next cultivated relatives. Metagenomic and metatranscriptomic analyses would provide even deeper insights into the functional potential of the community. However, 16S rRNA sequencing is less cost-intensive than whole metagenome shotgun sequencing and the investigation of the phylogenetic neighborhood already allows a hint toward the ecological role. Another possibility to predict functional profiles of microorganisms in the environment from 16S rRNA data is the usage of tools such as Tax4Fun (Aßhauer et al., 2015). Even though those functional predictions and our approach to investigate the next relatives cannot replace metagenomic or transcriptomic sequencing, it can still hint toward the role of microorganisms in the environment and consequently might provide helpful information to further cultivation approaches.

### Correlating the Occurrence of Individual OTUs to Environmental Settings Provide Additional Hints Concerning the Physiology of Uncultured *Rhodobacteraceae*

As our comprehensive dataset consists of bacterial 16S rRNA transcripts, it reflects the community composition of the metabolically active *Rhodobacteraceae* at the different sampling sites. As stated, the function of uncultured members of this family remains uncertain, if they are represented by OTUs that could not be affiliated to any cultured relatives, but linking their occurrence at a specific location to the respective environmental conditions can be used to predict some of their physiologic properties.

The *Rhodobacteraceae* communities generally grouped according to the individual sampling sites as indicated by the NMDS analysis. Here, the active communities of the shallow sites (max. water depth 50 mbsl) clustered together where the available carbon sources are supposed to be less recalcitrant, while at the sites exhibiting water depths of several thousand meters (e.g., North Pond, 4500 mbsl) the organic matter reaching the seafloor is already degraded during sedimentation (Martin et al., 1987; Karl et al., 1988). In contrast, the occurrence of individual OTUs neither correlated to the geographic location, nor to the water or sediment depth. Significant correlations (*p* ≤ 0.05) were identified for one-third of all active and detected *Rhodobacteraceae*, but mostly for only one single parameter related to the redox conditions in the investigated sediments. Most OTUs were significantly correlated to the sulfide concentrations in the porewater, suggesting that members of the respective genera thrive under anoxic conditions. Among the cultured representatives, these OTUs were affiliated to *Aestuariivita*, *Halocynthiibacter, Rubribacterium*, *Sagittula*, and *Salinihabitans.* However, anaerobic growth was only reported for the genus *Rubribacterium* (Boldareva et al., 2009), while the other four genera were described as aerobic. This contradictory result might be explained by the incomplete physiological characterization during the strain descriptions, as often nitrate reduction is the only anaerobic metabolism tested (e.g., Yoon et al., 2009; Kim et al., 2014; Park et al., 2014c). Our findings suggest that these genera might contain at least facultative anaerobic members. Thus, deposited isolates should physiologically be investigated in more detail to unravel their response to anoxic conditions or screen their genomes, if available. Novel phyla within the described genera that are not in culture yet could be capable of an anaerobic lifestyle. This approach might not be appropriate for those OTUs that are representative for so-far uncultured *Rhodobacteraceae* as no isolates are available. Detecting their 16S rRNA sequences within metagenomic datasets has the potential to shed light on their physiological properties (Streit and Schmitz, 2004). Furthermore, a directed molecular survey to search for their occurrence in the environment in combination with a molecular-guided isolation from respective enrichments would be the gold standard to identify their ecological function.

## Conclusion

By sharing, revisiting and reclassifying already existing datasets, we were able to analyze the metabolically active *Rhodobacteraceae* community compositions without redundant sample acquisitions. We could identify new clusters within this family and showed that a large part of the hidden diversity was particularly found in the subgroups other than the *Roseobacter* group. Their physiological properties might either be predicted by identifying the phylogenetic relationship of the respective OTUs or correlating their occurrence to environmental settings. Overall, our findings indicate that at least one-third of benthic *Rhodobacteraceae* was significantly correlated to the prevailing redox conditions (*p* ≤ 0.05). They are probably thriving under anoxic conditions and were thus not isolated using the common cultivation-based approaches.

## Data Availability

Datasets are in a publicly accessible repository. The 16S rRNA transcript nucleotide sequences have been deposited in the National Institutes of Health (NIH) GenBank database under BioProject numbers PRJNA527322 (Nueces River mouth), PRJNA526779 (Gulf of Mexico), PRJNA527340 (South Atlantic), PRJNA527344 (Equatorial Pacific), PRJNA308331 (North Pond), and Sequence Read Archive numbers SRP010369 (Gulf of Mexico) and SRA049352 (Nankai Trough).

## Author Contributions

MP and BE interpreted results and wrote the first draft of the manuscript. MP calculated phylogenetic trees and LD did the statistical data analyses. BW reprocessed the sequence raw data. BR and HM performed the sediment sampling during various expeditions and did extraction of nucleic acids and pyrosequencing. All authors were involved in critical revision and approval of the final version.

## Conflict of Interest Statement

HM was employed by Rhodium Scientific LLC. The remaining authors declare that the research was conducted in the absence of any commercial or financial relationships that could be construed as a potential conflict of interest.

## Footnotes

1 www.bacterio.net/-classifphyla.html

2 www.ncbi.nlm.nih.gov/Taxonomy/Browser/wwwtax.cgi

3 www.gbif.org/species/4729

4 www.bacterio.net/-classifphyla.html

5 www.ncbi.nlm.nih.gov/Taxonomy/Browser/wwwtax.cgi

6 www.gbif.org/species/4729

7 https://inkscape.org

8 http://www.oxfordjournals.org/our_journals/nar/database/a/

9 https://www.arb-silva.de/browser/

10 www.bacterio.net/-classifphyla.html
