# Supplementary material for: The Majority of Active Rhodobacteraceae in Marine Sediments Belong to Uncultured Genera: A Molecular Approach to Link Their Distribution to Environmental Conditions
Source: Front Microbiol. 2019 Apr 2;10:659. doi: 10.3389/fmicb.2019.00659 (PMC6454203; doi:10.3389/fmicb.2019.00659)
Supplement: Supplementary file 1 [file Data_Sheet_1.PDF]

Tab. S1 Overview of sampling sites, sorted by water depth, and respective environmental parameter.

Site	Expedition	Year of sampling	Water depth [mbsl]	Reference	Site name	Specification	Longitude	Latitude	Sediment depth	TOC content [%]	Sulfate [mM]	Sulfide [µM]	Fe^{2+} [µM]
**Nueces River mouth**	Field sampling ca	2009	3	Reese et al., 2014	1	Bay site, April	27°50.5440'N	97°24.6220'W	0 - 2 cmbsf	0.63	30.46	0	3.61
									2 - 4 cmbsf	0.4	24.87	0	1.31
									4 - 6 cmbsf	0.41	30.67	0	0
									6 - 8 cmbsf	0.53	25.82	0	0
						Bay site, September	27°50.5440'N	97°24.6220'W	0 - 2 cmbsf	0.54	56.65	0	4.3
									2 - 4 cmbsf	0.45	58.23	0	0
					3	River site, April	27°51.6250'N	97°33.3660'W	0 - 2 cmbsf	0.19	16.09	0.72	1.44
									2 - 4 cmbsf	0.51	18.84	132.78	0.64
									4 - 6 cmbsf	0.19	11.37	22.31	0
									6 - 8 cmbsf	0.29	3.44	259.86	0
						River site, September	27°51.6250'N	97°33.3660'W	0 - 2 cmbsf	1.15	2.98	139.17	4.41
									2 - 4 cmbsf	1.29	3.4	142.2	2.42
									4 - 6 cmbsf	0.96	1.56	156.46	3.09
									6 - 8 cmbsf	0.77	2.59	166.57	2.97
									8 - 10 cmbsf	1.22	4.87	184.44	0
**Gulf of Mexico**	MCH11	2008	20	Reese et al., 2013	8C		28°59.8560'N	92°0.3060'W	0 - 2 cmbsf	0.3	33.23	0	2.52
									2 - 4 cmbsf	0.46	30.33	0	1.62
									4 - 6 cmbsf	0.5	32.37	0	1.26
									6 - 8 cmbsf	0.57	30.33	0	0
									8 - 10 cmbsf	0.64	28.83	0	0
									10 - 12 cmbsf	0.49	31.51	0	NA
									12 - 14 cmbsf	0.57	NA	NA	NA
					BC1		28°52.8360'N	91°44.2380'W	0 - 2 cmbsf	0.93	35.16	0	NA
									2 - 4 cmbsf	0.93	30.01	0	NA
									4 - 6 cmbsf	0.95	28.73	0.01	NA
									6 - 8 cmbsf	0.89	28.73	0	NA
									8 - 10 cmbsf	0.71	29.91	0	NA
									10 - 12 cmbsf	0.74	29.58	0	NA
									12 - 14 cmbsf	0.73	30.12	0.37	NA
									14 - 16 cmbsf	0.63	26.05	0.02	NA
									16 - 18 cmbsf	0.61	28.3	NA	NA
**Gulf of Mexico**	MCH12	2008	20	Reese et al., 2013	8C		28°59.8560'N	92°0.3060'W	0 - 2 cmbsf	0.49	33.44	0	NA
									2 - 4 cmbsf	0.45	33.34	0.06	NA
									4 - 6 cmbsf	0.47	30.33	0	NA
									6 - 8 cmbsf	0.44	29.42	0.03	NA
									8 - 10 cmbsf	0.26	30.01	0	NA
									10 - 12 cmbsf	0.32	27.64	0	NA
									12 - 14 cmbsf	0.47	28.19	0	NA
									14 - 16 cmbsf	0.48	30.3	0	NA
									16 - 18 cmbsf	0.42	27.3	0	NA
									18 - 20 cmbsf	NA	29.16	0	NA
					BC1		28°52.8360'N	91°44.2380'W	0 - 2 cmbsf	0.75	30.66	0	NA
									2 - 4 cmbsf	0.64	28.51	0	NA
									4 - 6 cmbsf	0.57	26.8	0.54	NA
									6 - 8 cmbsf	0.33	34.19	1.28	NA
									8 - 10 cmbsf	0.44	30.44	2.31	NA
									10 - 12 cmbsf	0.34	30.55	0	NA
									12 - 14 cmbsf	0.78	29.8	0.21	NA
									14 - 16 cmbsf	0.76	28.4	0	NA
									16 - 18 cmbsf	0.61	30.44	0	NA
									18 - 20 cmbsf	0.64	30.44	0	NA

Continuation of Tab. S1

**Site**	**Expedition**	**Year of sampling**	**Water depth [mbsl]**	**Reference**	**Site name**	**Specification**	**Longitude**	**Latitude**	**Sediment depth**	**TOC content [%]**	**Sulfate [mM]**	**Sulfide [µM]**	**Fe^{2+} [µM]**
**Gulf of Mexico**	MCH14	2009	20	Reese, 2011	8C	noon	29°0.0240'N	92°0.2340'W	0 - 2 cmbsf	0.48	31.73	0.2	17.3
									2 - 4 cmbsf	0.7	34.04	0.5	27.07
									4 - 6 cmbsf	0.41	27.68	0.11	4.59
									6 - 8 cmbsf	0.53	24.35	0.07	0
									8 - 10 cmbsf	0.54	24.14	0	0.56
									10 - 12 cmbsf	0.55	26.35	0	0.92
									12 - 14 cmbsf	0.53	26.03	0	1.88
									14 - 16 cmbsf	0.53	21.63	0	0
									16 - 18 cmbsf	0.53	29.46	0	0
						midnight			0 - 2 cmbsf	0.36	25.26	0	9.41
									2 - 4 cmbsf	0.26	29.41	0	24.25
									4 - 6 cmbsf	0.61	24.86	0	21.74
									6 - 8 cmbsf	0.52	17.63	0	1.29
									8 - 10 cmbsf	0.55	14.41	0	0
									10 - 12 cmbsf	0.55	14	0	0.88
									12 - 14 cmbsf	0.52	12.56	0	0
									14 - 16 cmbsf	0.57	9.69	0	0
									16 - 18 cmbsf	0.54	27.77	0	0
					10B	noon	28°37.7400'N	90°33.0780'W	0 - 2 cmbsf	0.24	22.7	0	1.01
									2 - 4 cmbsf	0.36	26.8	0.18	12.22
									4 - 6 cmbsf	0.37	31.31	0.18	15.13
									6 - 8 cmbsf	0.47	22.14	0.08	0.83
									8 - 10 cmbsf	0.62	19.21	0.08	0.7
									10 - 12 cmbsf	0.35	11.32	0	0.63
									12 - 14 cmbsf	0.37	14.77	0	0
									14 - 16 cmbsf	0.39	18.58	0	0.71
									16 - 18 cmbsf	0.35	16.27	0	0
									18 - 20 cmbsf	0.36	15.06	0	0
						midnight			0 - 2 cmbsf	0.31	30.71	0	6.5
									2 - 4 cmbsf	0.24	27.84	0.14	0.78
									4 - 6 cmbsf	0.39	24.46	0	12.44
									6 - 8 cmbsf	0.48	19.57	0	2.51
									8 - 10 cmbsf	0.45	23.66	0	0
									10 - 12 cmbsf	0.4	27.91	0	0.76
									12 - 14 cmbsf	0.47	29.6	0	0
									14 - 16 cmbsf	0.34	29.37	0	0.8
									18 - 20 cmbsf	NA	NA	NA	NA
					AB5	noon	29°4.8060'N	89°56.9580'W	0 - 2 cmbsf	1.5	20.39	3.12	7.99
									2 - 4 cmbsf	1.46	16.13	0.34	23.6
									4 - 6 cmbsf	1.26	24.73	0.09	9.68
									6 - 8 cmbsf	1.09	17.83	0	0
									8 - 10 cmbsf	1.37	27.24	0	0
									10 - 12 cmbsf	1.42	14.81	0	0
									12 - 14 cmbsf	1.39	18.01	0	0
									14 - 16 cmbsf	0.86	19.54	0	0
									16 - 18 cmbsf	0.58	18.61	0.57	0
									18 - 20 cmbsf	0.58	28.14	0.45	0
						midnight			0 - 2 cmbsf	1.7	36.83	0.61	88.48
									2 - 4 cmbsf	1.45	14.53	0.96	109.51
									4 - 6 cmbsf	1.4	23.03	1	80.73
									6 - 8 cmbsf	1.28	31.4	0.47	3.05
									8 - 10 cmbsf	1.26	27.03	0.38	0.29
									10 - 12 cmbsf	1.38	14.54	1	0
									12 - 14 cmbsf	1.26	24.88	0.64	0
									14 - 16 cmbsf	1.47	32.21	0.24	0
									16 - 18 cmbsf	1.37	25.49	0.17	0
									18 - 20 cmbsf	0.87	19.96	0	0

Continuation of Tab. S1

Site	Expedition	Year of sampling	Water depth [mbsl]	Reference	Site name	Specification	Longitude	Latitude	Sediment depth	TOC content [%]	Sulfate [mM]	Sulfide [µM]	Fe^{2+} [µM]
**Gulf of Mexico**	MCH19	2011	20	Reese, 2011	8C	noon	28°59.8560'N	92°0.3060'W	0 - 1 cmbsf	NA	NA	NA	NA
									1 - 2 cmbsf	NA	NA	NA	NA
						midnight			0 - 1 cmbsf	NA	NA	NA	NA
									1 - 2 cmbsf	NA	NA	NA	NA
					10B	noon	28°37.7400'N	90°33.0780'W	0 - 1 cmbsf	NA	NA	NA	NA
									1 - 2 cmbsf	NA	NA	NA	NA
						midnight			0 - 1 cmbsf	NA	NA	NA	NA
									1 - 2 cmbsf	NA	NA	NA	NA
					AB5	noon	29°4.8060'N	89°56.9580'W	0 - 1 cmbsf	NA	NA	NA	NA
									1 - 2 cmbsf	NA	NA	NA	NA
						midnight			0 - 1 cmbsf	NA	NA	NA	NA
									1 - 2 cmbsf	NA	NA	NA	NA
**Gulf of Mexico**	MCH21	2012	20	Reese, 2011	8C	noon	28°59.8560'N	92°0.3060'W	0 - 1 cmbsf	NA	NA	NA	NA
									1 - 2 cmbsf	NA	NA	NA	NA
						midnight			0 - 1 cmbsf	NA	NA	NA	NA
									1 - 2 cmbsf	NA	NA	NA	NA
					10B	noon	28°37.7400'N	90°33.0780'W	0 - 1 cmbsf	NA	NA	NA	NA
									1 - 2 cmbsf	NA	NA	NA	NA
						midnight			0 - 1 cmbsf	NA	NA	NA	NA
									1 - 2 cmbsf	NA	NA	NA	NA
					AB5	noon	29°4.8060'N	89°56.9580'W	0 - 1 cmbsf	NA	NA	NA	NA
									1 - 2 cmbsf	NA	NA	NA	NA
						midnight			0 - 1 cmbsf	NA	NA	NA	NA
									1 - 2 cmbsf	NA	NA	NA	NA
**Palmyra Atoll**	Field sampling ca	2009	50	Unpublished	1	East	5°52.9033'N	162°3.6083'W	0 - 1 cmbsf	NA	NA	NA	NA
						West	5°53.2916'N	162°5.9783'W	0 - 1 cmbsf	NA	NA	NA	NA
					2	East	5°53.2150'N	162°3.6950'W	0 - 1 cmbsf	NA	NA	NA	NA
						West	5°53.1583'N	162°5.2633'W	0 - 1 cmbsf	NA	NA	NA	NA
					3	East	5°53.0816'N	162°3.7500'W	0 - 1 cmbsf	NA	NA	NA	NA
						West	5°52.9667'N	162°5.9433'W	0 - 1 cmbsf	NA	NA	NA	NA
					Central		5°53.1300'N	162°4.3717'W	0 - 1 cmbsf	NA	NA	NA	NA
			10		Outer		5°52.7000'N	162°3.3783'W	0 - 1 cmbsf	NA	NA	NA	NA
					Inner		5°52.7233'N	162°3.7350'W	0 - 1 cmbsf	NA	NA	NA	NA
**Nankai Trough**	IODP 316	2007/2008	2627	Mills et al., 2012	C0004	1H1	33°13.0000'N	136°43.0000'E	1 mbsf	0.46	25.3	NA	2.5
						3H4			20 mbsf	0.42	0.86	NA	0
			3100		8A		33°12.8229'N	136°43.5997'E	2.16 mbsf	0.7	23.86	NA	2.48
									7.26 mbsf	0.65	2.27	NA	1.51
**South Atlantic**	M78/3	2009	3687	Unpublished	GeoB 13863		39°18.7000'S	53°57.1600'W	8.43 mbsf	0.8	NA	NA	NA
**Equatorial Pacific**	IODP 320	2009	4200	Unpublished	U1332A		2°30.4690'N	117°58.1780'W	7.55 mbsf	0	27.6	NA	0
									26.55 mbsf	0.12	27.9	NA	0
**North Pond**	IODP 336	2011	4500	Reese et al., 2018	U1382B		22°45.0000'N	46°5.0000'W	4.5 mbsf	NA	NA	NA	NA
									46.5 mbsf	NA	NA	NA	NA
					U1383D		22°48.0000'N	46°3.0000'W	8.5 mbsf	NA	NA	NA	NA
									26.0 mbsf	NA	NA	NA	NA
									38.5 mbsf	NA	NA	NA	NA

Mills, H.J., Reese, B.K., Shepard, A.K., Riedinger, N., Dowd, S.E., Morono, Y., and Inagaki, F. (2012). Characterization of metabolically active bacterial populations in subseafloor Nankai Trough sediments above, within, and below the sulfate-methane transition zone. *Frontiers in Microbiology* 3, 12. doi: 10.3389/fmicb.2012.00113.

Reese, B.K. (2011). Linking molecular microbiology and geochemistry to better understand microbial ecology in coastal marine sediments. *Dissertation at Texas A&M University.*

Reese, B.K., Mills, H.J., Dowd, S.E., and Morse, J.W. (2013). Linking molecular microbial ecology to geochemistry in a coastal hypoxic zone. *Geomicrobiology Journal* 30, 160-172. doi: 10.1080/01490451.2012.659331.

Reese, B.K., Witmer, A.D., Moller, S., Morse, J.W., and Mills, H.J. (2014). Molecular assays advance understanding of sulfate reduction despite cryptic cycles. *Biogeochemistry* 118**,** 307-319. doi: 10.1007/s10533-013-9933-2.

Reese, B.K., Zinke, L.A., Sobol, M.S., LaRowe, D.E., Orcutt, B.N., Zhang, X., Jaekel, U., Wang, F., Dittmar, T., Defforey, D., Tully, B., Paytan, A., Sylvan, J.B., Amend, J.P., Edwards, K.J., and Girguis, P. (2018). Nitrogen cycling of active bacteria within oligotrophic oligotrophic sediment of the Mid-Atlantic Ridge flank. *Geomicrobiology Journal* 35, 6. doi: 10.1080/01490451.2017.1392649.
