# Supplementary material for: The Majority of Active Rhodobacteraceae in Marine Sediments Belong to Uncultured Genera: A Molecular Approach to Link Their Distribution to Environmental Conditions
Source: Front Microbiol. 2019 Apr 2;10:659. doi: 10.3389/fmicb.2019.00659 (PMC6454203; doi:10.3389/fmicb.2019.00659)
Supplement: Supplementary file 2 [file Data_Sheet_2.PDF]

Tab. S2 Relative abundance of *Rhodobacteraceae* -OTUs on the total bacterial community [%] in the depth-clustered sites. The affiliation of OTUs to the phylogenetic subgroups is displayed by red = *Roseobacter* , orange = *Stappia* , green = *Amaricoccus* , purple = *Rhodobacter* and blue = *Paracoccus* .

Sampling site	Nueces River mouth					Gulf of Mexico										Palmyra Atoll	Nankai Trough		South Atlantic	Equatorial Pacific		North Pond	
Depth	0 - 2	2 - 4	4 - 6 cmbsf	6 - 8	8 - 10	0 - 2	2 - 4	4 - 6	6 - 8	8 - 10 cmbsf	10 - 12	12 - 14	14 - 16	16 - 18	18 - 20	0 - 2 cmbsf	0.2 - 10 mbsf	10 - 20	0.2 - 10 mbsf	0.2 - 10 mbsf	20 - 100	0.2 - 10 mbsf	20 - 100
Actibacterium	0.006	0	0	0.005	0	0.023	0.008	0.015	0.004	0	0	0.212	0.013	0	0	0.024	0	0	0	0	0	0	0
Aestuariivita	0.002	0	0	0	0	0	0	0	0	0	0	0	0	0	0	0	0	0	0	0	0	0	0
Amaricoccus	0	0	0	0	0	0	0	0	0	0	0	0	0.365	0	0	0	0	0	0	0	0	0	0
Amylibacter	0	0	0	0	0	0	0	0	0	0	0	0	0	0.006	0	0	0	0	0	0	0	0	0
Ascidiaceihabitans	0.003	0	0	0	0	0.006	0	0	0.004	0	0	0	0	0	0.004	0	0	0	0	0	0	0	0
Boseongicola	0.006	0	0	0	0	0.006	0	0	0	0	0	0	0	0	0	0.007	0	0	0	0	0	0	0
CHAB-I-5 lineage	0.003	0	0	0	0	0.051	0.004	0.004	0.004	0.003	0	0.424	0	0.006	0	0	0	0	0	0	0	0	0
Defluviimonas	0.014	0	0.008	0	0	0.011	0.004	0	0	0	0	0	0	0	0	0	0	0	0	0	0	0	0
Dinoroseobacter	0	0	0	0	0	0.101	0	0	0.004	0	0	0	0.025	0.019	0	0.038	0	0	0	0	0	0	0
Epibacterium	0	0	0	0	0	0	0	0	0	0.007	0	0	0	0	0	0	0	0	0	0	0	0	0
Halocynthiibacter	0.002	0	0	0	0	0	0	0	0	0	0	0	0	0	0	0	0	0	0	0	0	0	0
Labrenzia	0.002	0	0	0	0	0.011	0	0	0	0	0	0	0	0	0	0	0	0	0	0	0	0	0
Leisingera	0	0	0	0	0	0	0	0	0.004	0	0	0	0	0	0	0.003	0	0	0	0	0	0	0
Lentibacter	0	0	0	0	0	0.006	0	0	0	0.003	0	0	0	0	0	0	0	0	0	0	0	0	0
Litorimicrobium	0.002	0	0	0	0	0.011	0	0	0	0	0	0	0	0	0	0.003	0	0	0	0	0	0	0
Loktanella	0.002	0	0	0	0	0.006	0	0	0	0	0	0	0	0	0	0.007	0	0	0	0	0	0	0
Marivita	0	0	0	0	0	0.045	0	0.004	0	0.003	0	0	0	0	0	0.007	0	0	0	0	0	0	0
Oceanicella	0.003	0	0	0.009	0	0	0.004	0.004	0.004	0.003	0	0	0	0	0	0.017	0	0	0	0	0	0	0
Oceanobacterium	0	0	0	0	0	0	0	0	0	0	0	0	0	0	0	0.007	0	0	0	0	0	0	0
Paracoccus	0	0	0	0	0	0	0.004	0	0	0	0	0	0	0	0	0	1.465	0	0	0	0	0	3.766
Phaeomarinomonas	0	0	0	0	0	0.141	0.033	0.037	0.009	0.037	0.004	0.212	0.013	0.025	0	0	0	0	0	0	0	0	0
Planktomarina	0	0	0	0	0	0.039	0.004	0	0	0	0	0	0	0	0.004	0	0	0	0	0	0	0	0
Planktotalea	0	0	0	0	0	0	0	0	0	0	0	0	0	0	0	0.003	0	0	0	0	0	0	0
Pseudophaeobacter	0	0	0	0	0	0.006	0	0	0	0	0	0.212	0	0	0	0	0	0	0	0	0	0	0
Pseudoroseovarius	0	0	0	0	0	0.006	0	0	0	0.003	0	0	0	0	0	0	0	0	0	0	0	0	1.255
Pseudoruegeria	0	0	0	0	0	0.073	0.021	0.007	0	0.003	0	0.636	0	0	0	0.007	0	0	0	0	0	0	0
Roseibacterium	0.012	0.003	0	0.009	0	0	0	0	0	0	0	0	0	0	0	0	0	0	0	0	0	0	0
Roseibium	0	0	0	0	0	0.006	0	0	0	0	0	0	0	0	0	0	0	0	0	0	0	0	0
Roseobacter	0	0	0	0	0	0.006	0.004	0	0.004	0	0	0	0	0	0	0.007	0	0	0	0	0	0	0
NAC11-7 lineage	0.003	0	0	0	0	0.011	0	0	0	0	0	0	0	0	0	0.007	0	0	0	0	0	0	0
Roseovarius	0.008	0	0	0.005	0.014	0.011	0.004	0	0.004	0.003	0	0	0	0	0	0.065	0.039	0	0	0	0	0	0
Rubellimicrobium	0.002	0	0	0	0	0	0	0	0.009	0	0.073	0	0.101	0	0	0	0	0	0	0	0.010	0	0
Rubribacterium	0.014	0	0	0	0	0	0	0	0	0	0	0	0	0	0	0.007	0	0	0	0	0	0	0
Rubrimonas	0.003	0	0	0	0	0	0	0	0	0	0	0	0	0	0	0	0	0	0	0	0	0	0
Ruegeria	0.002	0	0	0	0	0.124	0.012	0.011	0.013	0	0	0.212	0	0	0	0.017	0	0	0	0	0	0	0
Sagittula	0.003	0	0	0	0	0	0	0	0	0	0	0	0	0	0	0	0	0	0	0	0	0	0
Salinihabitans	0	0	0	0	0.362	0	0	0	0	0	0	0	0	0	0	0	0	0	0	0	0	0	0
Shimia	0	0	0	0	0	0.056	0	0	0	0	0	0	0	0	0	0.007	0	0.004	0	0	0	0	0
Stappia	0	0	0	0	0	0	0	0	0	0	0	0	0	0	0	0	0	0	0	0	0	0	3.766
Sulfitobacter	0	0	0	0	0	0	0	0	0	0	0	0	0	0	0	0	0	0	0.055	0	0	2.719	0
Tateyamaria	0.002	0	0	0	0	0	0	0	0	0	0	0	0	0	0	0	0	0	0	0	0	0	0
Thalassobius	0	0	0	0	0	0.107	0.012	0.007	0.009	0.003	0.012	0	0	0	0.007	0.007	0	0	0	0	0	0	0
Tropicibacter	0	0	0	0.019	0	0.011	0	0	0	0	0	0	0	0	0	0.003	0	0.004	0	0	0	0	0
Tropicimonas	0.005	0	0	0	0	0.023	0.004	0	0	0	0	0	0.013	0	0	0.120	0.154	0	0	0	0	0	0
**uncultured_1**	0.002	0	0	0	0	0	0	0	0.004	0	0	0	0	0	0	0	0	0	0	0	0	0	0
**uncultured_2**	0	0	0	0.009	0	0	0	0	0	0	0	0	0	0	0	0	0	0	0	0	0	0	0
**uncultured_3**	0	0	0	0.009	0	0	0	0	0	0	0	0	0	0	0	0	0	0	0	0	0	0	0
**uncultured_4**	0.006	0	0	0.009	0	0	0	0	0	0	0	0	0	0	0	0	0	0	0	0	0	0	0
**uncultured_5**	0	0	0	0.033	0	0	0	0	0	0	0	0	0	0	0	0	0	0	0	0	0	0	0
**uncultured_6**	0.015	0	0.004	0.014	0	0	0	0	0	0	0	0	0	0	0	0	0	0	0	0	0	0	0
**uncultured_7**	0.020	0	0.032	0.009	0	0	0	0	0	0	0	0	0	0	0	0	0	0	0	0	0	0	0
**uncultured_8**	0	0	0	0	0	0	0	0	0	0	0	0	0	0	0	0.003	0	0	0	0	0	0	0
**uncultured_9**	0	0	0.004	0	0	0	0	0	0	0	0	0	0	0	0	0	0	0	0	0	0	0	0

Continuation of Tab. S2

Sampling site	Nueces River mouth					Gulf of Mexico										Palmyra Atoll	Nankai Trough		South Atlantic	Equatorial Pacific		North Pond	
Depth	0 - 2	2 - 4	4 - 6	6 - 8	8 - 10	0 - 2	2 - 4	4 - 6	6 - 8	8 - 10	10 - 12	12 - 14	14 - 16	16 - 18	18 - 20	0 - 2	0.2 - 10	10 - 20	0.2 - 10	0.2 - 10	20 - 100	0.2 - 10	20 - 100
	cmbsf					cmbsf										cmbsf	mbsf		mbsf	mbsf		mbsf	
**uncultured_10**	0.003	0	0.004	0	0	0	0	0	0	0	0	0	0	0	0	0	0	0	0	0	0	0	0
**uncultured_11**	0.005	0	0	0	0	0	0	0	0	0	0	0	0	0	0	0	0	0	0	0	0	0	0
**uncultured_12**	0.002	0	0.012	0	0	0	0	0	0	0	0	0	0	0	0	0	0	0	0	0	0	0	0
**uncultured_13**	0.002	0	0.008	0	0	0	0.004	0	0	0	0	0	0	0	0	0.003	0	0	0	0	0	0	0
**uncultured_14**	0.020	0	0.106	0.023	0	0	0	0	0	0	0	0	0	0	0	0.007	0	0	0	0	0	0	0
**uncultured_15**	0	0	0	0	0	0.006	0	0	0	0	0	0	0	0	0	0	0	0	0	0	0	0	0
**uncultured_16**	0	0	0	0	0	0	0	0	0	0	0	0	0	0	0	0.007	0	0	0	0	0	0	0
**uncultured_17**	0	0	0	0.005	0	0	0	0	0	0	0	0	0	0	0	0	0	0	0	0	0	0	0
**uncultured_18**	0	0	0	0.005	0	0	0	0.004	0	0	0	0	0	0	0	0	0	0	0	0	0	0	0
**uncultured_19**	0	0	0	0	0	0	0	0	0	0	0	0	0	0	0	0.024	0	0	0	0	0	0	0
**uncultured_20**	0	0	0	0	0	0	0	0	0	0	0.004	0	0	0	0	0	0	0	0	0	0	0	0
**uncultured_21**	0	0	0	0	0	0.006	0	0	0	0	0	0	0	0	0	0	0	0	0	0	0	0	0
**uncultured_22**	0.002	0	0	0	0	0	0	0	0	0	0	0	0	0	0	0	0	0	0	0	0	0	0
**uncultured_23**	0.002	0	0	0	0	0	0	0	0	0	0	0	0	0	0	0	0	0	0	0	0	0	0
**uncultured_24**	0	0	0	0	0	0	0.004	0	0	0	0	0	0	0	0	0	0	0	0	0	0	0	0
**uncultured_25**	0	0	0	0	0	0	0	0	0	0	0	0	0	0	0	0	0.013	0	0	0	0	0	0
**uncultured_26**	0	0	0	0	0	0	0	0	0	0	0	0	0.013	0.006	0	0	0	0	0	0	0	0	0
**uncultured_27**	0	0	0	0	0	0	0.004	0	0	0	0	0.212	0	0	0	0	0	0	0	0	0	0	0
**uncultured_28**	0	0	0	0	0	0.011	0	0	0	0	0	0	0	0	0	0	0	0	0	0	0	0	0
**uncultured_29**	0	0	0	0	0	0	0.004	0	0.004	0	0	0	0	0	0	0	0	0	0	0	0	0	0
**uncultured_30**	0	0	0	0	0	0	0	0.004	0.004	0	0	0	0	0	0	0	0	0	0	0	0	0	0
**uncultured_31**	0.002	0	0	0	0	0.006	0	0	0	0	0	0	0	0	0	0	0	0	0	0	0	0	0
**uncultured_32**	0	0	0	0	0	0.006	0	0	0.004	0	0	0	0	0	0	0	0	0	0	0	0	0	0
**uncultured_33**	0	0	0	0	0	0	0	0	0	0	0	0	0	0	0	0.007	0	0	0	0	0	0	0
**uncultured_34**	0.003	0	0	0	0	0	0	0	0	0	0	0	0	0	0	0	0	0	0	0	0	0	0
**uncultured_35**	0.003	0	0	0	0	0	0	0	0	0	0	0	0	0	0	0	0	0	0	0	0	0	0
**uncultured_36**	0	0	0	0	0	0.006	0	0.004	0	0	0	0	0	0	0	0	0	0	0	0	0	0	0
**uncultured_37**	0	0	0	0	0	0	0	0	0	0	0	0.636	0	0	0	0	0	0	0	0	0	0	0
**uncultured_38**	0	0	0	0	0	0	0	0	0	0	0	0	0	0	0	0.010	0	0	0	0	0	0	0
**uncultured_39**	0	0	0	0	0	0	0	0	0	0	0	0	0	0	0	0.010	0	0	0	0	0	0	0
**uncultured_40**	0	0	0	0	0	0	0.004	0	0	0	0	0.212	0.013	0	0	0	0	0	0	0	0	0	0
**uncultured_41**	0	0	0	0	0	0.017	0	0	0	0	0	0	0	0	0	0	0	0	0	0	0	0	0
**uncultured_42**	0	0	0	0	0	0	0	0	0	0	0	0	0	0	0	0.010	0	0	0	0	0	0	0
**uncultured_43**	0.005	0	0	0	0	0	0	0	0	0	0	0	0	0	0	0	0	0	0	0	0	0	0
**uncultured_44**	0	0	0	0	0	0	0	0	0	0	0	0	0	0	0	0.010	0	0	0	0	0	0	0
**uncultured_45**	0	0	0	0	0	0	0	0.004	0	0.007	0	0	0	0	0	0	0	0	0	0	0	0	0
**uncultured_46**	0	0	0	0	0	0	0	0	0	0	0.008	0	0.013	0	0	0	0	0	0	0	0	0	0
**uncultured_47**	0	0	0	0	0	0.017	0	0	0	0	0	0	0	0	0.004	0	0	0	0	0	0	0	0
**uncultured_48**	0	0	0	0	0	0	0	0	0	0	0	0	0	0	0	0.014	0	0	0	0	0	0	0
**uncultured_49**	0.006	0	0	0	0	0	0	0	0	0	0	0	0	0	0	0	0	0	0	0	0	0	0
**uncultured_50**	0	0	0	0	0	0	0	0.004	0.009	0	0.004	0.212	0	0	0	0.003	0	0	0	0	0	0	0
**uncultured_51**	0	0	0	0	0	0.006	0	0.004	0.004	0.003	0.004	0.424	0	0	0	0	0	0	0	0	0	0	0
**uncultured_52**	0.009	0	0	0	0	0.011	0	0	0	0	0	0	0	0	0	0	0	0	0	0	0	0	0
**uncultured_53**	0	0.027	0	0	0	0	0	0	0	0	0	0	0	0	0	0	0	0	0	0	0	0	0
**uncultured_54**	0	0	0	0	0	0	0	0	0	0.010	0	0.636	0.025	0	0	0.007	0	0	0	0	0	0	0
**uncultured_55**	0	0	0	0	0	0.011	0	0.004	0	0.003	0	0	0.025	0.025	0	0	0	0	0	0	0	0	0
**uncultured_56**	0	0	0	0	0	0	0	0	0	0	0	0	0	0	0	0.041	0.013	0	0	0	0	0	0
**uncultured_57**	0	0	0	0	0	0.006	0	0.004	0.031	0.003	0.004	0	0	0	0	0	0.026	0	0	0	0	0	0
**uncultured_58**	0.015	0	0	0	0	0	0	0.004	0.004	0	0	0	0	0	0	0.014	0.013	0	0	0	0	0	0
**uncultured_59**	0.023	0	0	0.014	0	0.006	0	0	0	0	0	0	0	0	0	0	0	0	0	0	0	0	0
**uncultured_60**	0	0	0	0	0	0.023	0	0.007	0.013	0.010	0.004	0	0.025	0.013	0.007	0	0	0	0	0	0	0	0
**uncultured_61**	0	0	0	0	0	0	0	0	0.004	0	0	0	0	0	0	0.082	0.026	0	0	0	0	0	0
**uncultured_62**	0.008	0.013	0	0.075	0	0	0.004	0.007	0.004	0.010	0.004	0	0	0	0	0	0	0	0	0	0	0	0
**uncultured_63**	0	0	0	0	0	0.068	0.021	0.011	0.013	0.017	0.020	0	0	0.019	0	0	0	0	0	0	0	0	0
**uncultured_64**	0	0	0	0	0	0.039	0.012	0.011	0.031	0.030	0	0.424	0.025	0	0.004	0.007	0	0	0	0	0	0	0

Continuation of Tab. S2

Sampling site	Nueces River mouth					Gulf of Mexico										Palmyra Atoll	Nankai Trough		South Atlantic	Equatorial Pacific		North Pond	
**Depth**	0 - 2	2 - 4	4 - 6	6 - 8	8 - 10	0 - 2	2 - 4	4 - 6	6 - 8	8 - 10	10 - 12	12 - 14	14 - 16	16 - 18	18 - 20	0 - 2	0.2 - 10	10 - 20	0.2 - 10	0.2 - 10	20 - 100	0.2 - 10	20 - 100
	cmbsf					cmbsf										cmbsf	mbsf		mbsf	mbsf		mbsf	
**uncultured_65**	0	0	0	0	0	0.073	0.012	0.015	0.013	0.037	0.029	0.847	0.038	0.019	0.004	0	0	0	0	0	0	0	0
**uncultured_66**	0.035	0	0.012	0.061	0	0.034	0.008	0.011	0.013	0.003	0.012	0.424	0	0	0	0	0	0	0	0	0	0	0
**uncultured_67**	0	0	0	0	0	0.118	0.016	0.030	0.013	0.017	0.029	1.695	0.063	0.006	0.004	0	0	0	0	0	0	0	0
**uncultured_68**	0.003	0	0	0.089	0	0.056	0.004	0.022	0.018	0.044	0.012	0	0	0.019	0	0.024	0	0	0	0	0	0	0
**uncultured_69**	0.018	0.201	0	0	0	0.011	0	0	0	0	0	0	0	0	0	0	0	0	0	0	0	0	0
**uncultured_70**	0	0	0	0	0	0	0	0	0	0	0	0	0	0	0	0.236	0.077	0	0	0	0	0	0
**uncultured_71**	0	0	0	0	0	0	0	0	0	0	0	0	0	0	0	0	0	0	0	0	0	1.108	14.644
**uncultured_72**	0	0	0	0	0	0.006	0	0	0	0	0	0	0	0	0	0	0	0	0	0	0	0	0
**uncultured_73**	0.002	0	0	0	0	0	0	0	0	0	0	0	0	0	0	0	0	0	0	0	0	0	0
**uncultured_74**	0	0	0	0	0	0	0	0.004	0	0	0	0	0	0	0	0	0	0	0	0	0	0	0
**uncultured_75**	0	0	0	0	0	0	0.004	0	0	0	0	0	0	0	0	0	0	0	0	0	0	0	0
**uncultured_76**	0	0	0	0	0.005	0	0	0	0	0	0	0	0	0	0	0	0	0	0	0	0	0	0
**uncultured_77**	0	0	0	0	0	0	0	0	0	0	0	0	0.013	0	0	0	0	0	0	0	0	0	0
**uncultured_78**	0.002	0	0	0	0	0	0	0	0	0	0	0	0	0	0	0	0	0	0	0	0	0	0
**uncultured_79**	0.002	0	0	0	0	0	0	0	0	0	0	0	0	0	0	0	0	0	0	0	0	0	0
**uncultured_80**	0	0	0	0	0	0.006	0	0	0	0	0	0	0	0	0	0	0	0	0	0	0	0	0
**uncultured_81**	0.002	0	0	0	0	0	0	0	0	0	0	0	0	0	0	0	0	0	0	0	0	0	0
**uncultured_82**	0	0	0	0	0	0	0	0	0	0	0	0	0	0	0	0.003	0	0	0	0	0	0	0
**uncultured_83**	0	0	0	0.005	0	0	0	0	0	0	0	0	0	0	0	0	0	0	0	0	0	0	0
**uncultured_84**	0	0	0	0	0	0	0	0.004	0	0	0	0	0.013	0	0	0	0	0	0	0	0	0	0
**uncultured_85**	0	0	0	0	0	0	0	0	0	0	0.004	0.212	0	0	0	0	0	0	0	0	0	0	0
**uncultured_86**	0	0	0	0.009	0	0	0	0	0	0	0	0	0	0	0	0	0	0	0	0	0	0	0
**uncultured_87**	0	0	0	0	0	0.006	0	0	0	0	0	0.212	0	0	0	0	0	0	0	0	0	0	0
**uncultured_88**	0.002	0	0	0	0	0	0	0.004	0	0	0	0	0	0	0	0	0	0	0	0	0	0	0
**uncultured_89**	0	0	0	0	0	0.011	0	0	0	0	0	0	0	0	0	0	0	0	0	0	0	0	0
**uncultured_90**	0	0	0	0	0	0	0.004	0	0	0	0	0	0.013	0	0	0	0	0	0	0	0	0	0
**uncultured_91**	0.003	0	0	0	0	0	0	0	0	0	0	0	0	0	0	0	0	0	0	0	0	0	0
**uncultured_92**	0	0	0	0	0	0	0	0.004	0	0	0	0	0	0.006	0	0	0	0	0	0	0	0	0
**uncultured_93**	0	0	0	0	0	0	0	0	0.004	0	0	0	0	0	0	0.003	0	0	0	0	0	0	0
**uncultured_94**	0	0	0	0	0	0.006	0	0	0	0	0	0	0.013	0	0	0	0	0	0	0	0	0	0
**uncultured_95**	0	0	0	0	0	0	0	0	0	0	0	0	0	0	0	0.007	0	0	0	0	0	0	0
**uncultured_96**	0	0	0	0	0	0	0	0.004	0.004	0	0	0	0	0	0	0	0	0	0	0	0	0	0
**uncultured_97**	0	0	0	0	0	0	0	0	0	0	0	0	0	0.013	0	0	0	0	0	0	0	0	0
**uncultured_98**	0	0	0	0	0	0	0.004	0	0.004	0	0	0	0	0	0	0	0	0	0	0	0	0	0
**uncultured_99**	0	0	0	0	0	0	0.004	0	0	0.003	0	0	0	0	0	0	0	0	0	0	0	0	0
**uncultured_100**	0	0	0	0	0	0	0	0	0	0.003	0	0.212	0	0	0	0	0	0	0	0	0	0	0
**uncultured_101**	0	0	0	0	0	0.006	0.004	0	0.004	0	0	0	0	0	0	0	0	0	0	0	0	0	0
**uncultured_102**	0	0	0	0	0	0.006	0.008	0	0	0	0	0	0	0	0	0	0	0	0	0	0	0	0
**uncultured_103**	0	0	0	0	0	0	0.004	0	0.004	0	0	0	0	0	0.004	0	0	0	0	0	0	0	0
**uncultured_104**	0	0	0	0	0	0	0	0	0.004	0	0	0.212	0	0.006	0	0	0	0	0	0	0	0	0
**uncultured_105**	0	0	0	0	0	0	0	0	0	0	0	0	0	0	0	0.007	0.013	0	0	0	0	0	0
**uncultured_106**	0	0	0	0	0	0	0.004	0.004	0	0.003	0	0	0	0	0	0	0	0	0	0	0	0	0
**uncultured_107**	0	0	0	0	0	0.011	0	0	0	0	0	0	0	0.006	0	0	0	0	0	0	0	0	0
**uncultured_108**	0	0	0	0	0	0	0	0	0.004	0	0.004	0.212	0	0	0	0	0	0	0	0	0	0	0
**uncultured_109**	0.002	0	0.004	0	0	0	0	0	0	0	0	0	0	0	0	0.003	0	0	0	0	0	0	0
**uncultured_110**	0	0	0	0	0	0.011	0	0	0	0.003	0	0	0	0	0	0	0	0	0	0	0	0	0
**uncultured_111**	0	0	0	0	0	0	0.004	0	0	0	0	0.424	0	0	0	0	0	0	0	0	0	0	0
**uncultured_112**	0	0	0	0	0	0	0	0	0	0	0.004	0	0	0	0	0.007	0	0	0	0	0	0	0
**uncultured_113**	0	0	0	0	0	0.017	0	0	0	0	0	0	0	0	0	0	0	0	0	0	0	0	0
**uncultured_114**	0	0	0	0	0	0	0	0	0.004	0	0	0	0	0	0	0.007	0	0	0	0	0	0	0
**uncultured_115**	0	0	0	0	0	0.006	0	0	0	0	0	0	0.025	0	0	0	0	0	0	0	0	0	0
**uncultured_116**	0	0	0	0	0	0	0	0	0	0	0	0	0	0	0	0.010	0	0	0	0	0	0	0
**uncultured_117**	0	0	0	0.014	0	0	0	0	0	0	0	0	0	0	0	0	0	0	0	0	0	0	0
**uncultured_118**	0	0	0	0	0	0	0	0.004	0	0	0.004	0.212	0	0	0	0	0	0	0	0	0	0	0
**uncultured_119**	0	0	0	0	0	0.006	0	0	0	0	0.008	0	0	0	0	0	0	0	0	0	0	0	0

Continuation of Tab. S2

Sampling site	Nueces River mouth					Gulf of Mexico										Palmyra Atoll	Nankai Trough		South Atlantic	Equatorial Pacific		North Pond	
Depth	0 - 2	2 - 4	4 - 6	6 - 8	8 - 10	0 - 2	2 - 4	4 - 6	6 - 8	8 - 10	10 - 12	12 - 14	14 - 16	16 - 18	18 - 20	0 - 2	0.2 - 10	10 - 20	0.2 - 10	0.2 - 10	20 - 100	0.2 - 10	20 - 100
	cmbsf					cmbsf										cmbsf	mbsf		mbsf	mbsf		mbsf	
**uncultured_120**	0	0	0	0	0	0	0	0	0	0.003	0	0.424	0	0	0	0	0	0	0	0	0	0	0
**uncultured_121**	0	0.013	0	0	0	0	0	0	0	0	0	0	0	0	0	0	0	0	0	0	0	0	0
**uncultured_122**	0	0	0	0	0	0	0	0	0	0	0	0	0	0	0	0.014	0	0	0	0	0	0	0
**uncultured_123**	0.005	0	0	0	0	0.006	0	0	0	0	0	0	0	0	0	0	0	0	0	0	0	0	0
**uncultured_124**	0	0	0	0	0	0	0	0	0	0.003	0	0.212	0	0.013	0	0	0	0	0	0	0	0	0
**uncultured_125**	0	0	0	0	0	0	0	0	0.004	0	0	0	0	0.019	0	0	0	0	0	0	0	0	0
**uncultured_126**	0	0	0	0	0	0	0.004	0.004	0	0	0	0.212	0	0	0	0.003	0	0	0	0	0	0	0
**uncultured_127**	0	0	0	0	0	0	0	0.007	0.004	0	0.004	0	0	0	0	0	0	0	0	0	0	0	0
**uncultured_128**	0	0	0	0	0	0	0	0	0.004	0	0	0	0	0	0.011	0	0	0	0	0	0	0	0
**uncultured_129**	0	0	0	0	0	0	0	0	0	0	0	0	0	0	0	0.014	0	0	0	0	0	0	0
**uncultured_130**	0	0	0	0	0	0	0	0	0	0	0	0	0	0	0	0	0.051	0	0	0	0	0	0
**uncultured_131**	0	0	0	0	0	0.006	0	0	0.004	0	0	0	0	0	0.011	0	0	0	0	0	0	0	0
**uncultured_132**	0.002	0	0	0.009	0	0.011	0	0	0	0	0	0	0	0	0	0	0	0	0	0	0	0	0
**uncultured_133**	0	0	0	0	0	0	0	0	0	0	0.004	0	0	0	0.004	0.010	0	0	0	0	0	0	0
**uncultured_134**	0	0	0	0	0	0.011	0.004	0	0.004	0	0.004	0	0	0	0	0	0	0	0	0	0	0	0
**uncultured_135**	0	0	0	0	0	0.006	0.004	0	0	0.007	0	0	0.013	0	0	0	0	0	0	0	0	0	0
**uncultured_136**	0	0	0	0	0	0.006	0.004	0.004	0	0	0	0	0	0	0	0.007	0	0	0	0	0	0	0
**uncultured_137**	0	0	0	0	0	0	0	0	0	0	0	0	0	0	0	0.017	0	0	0	0	0	0	0
**uncultured_138**	0.006	0	0	0	0	0.006	0	0	0	0	0.004	0	0	0	0	0	0	0	0	0	0	0	0
**uncultured_139**	0.002	0	0	0	0	0.023	0.004	0	0	0	0	0	0	0	0	0	0	0	0	0	0	0	0
**uncultured_140**	0	0	0	0	0	0	0	0	0	0	0.008	0	0.050	0	0	0	0	0	0	0	0	0	0
**uncultured_141**	0	0	0	0	0	0.023	0	0	0	0	0	0	0	0	0	0.007	0	0	0	0	0	0	0
**uncultured_142**	0	0	0	0	0	0.006	0.004	0.004	0	0.007	0.004	0.212	0	0	0	0	0	0	0	0	0	0	0
**uncultured_143**	0	0	0	0	0	0	0	0	0	0	0.004	0	0	0	0	0.021	0	0	0	0	0	0	0
**uncultured_144**	0	0	0	0	0	0.011	0	0	0	0.003	0.004	0	0.038	0	0	0	0	0	0	0	0	0	0
**uncultured_145**	0	0	0	0	0	0	0	0	0	0	0	0	0	0	0	0.007	0.064	0	0	0	0	0	0
**uncultured_146**	0	0	0	0	0	0	0	0.004	0.004	0.003	0.004	0.212	0.013	0	0.007	0	0	0	0	0	0	0	0
**uncultured_147**	0	0	0	0	0	0	0.008	0.004	0	0.003	0.004	0	0.025	0	0.004	0	0	0	0	0	0	0	0
**uncultured_148**	0	0	0	0	0	0.006	0	0.007	0.009	0	0.004	0.424	0.013	0	0	0	0	0	0	0	0	0	0
**uncultured_149**	0	0	0	0	0	0	0	0	0	0	0	0	0	0	0	0.031	0	0	0	0	0	0	0
**uncultured_150**	0.003	0	0	0	0	0.017	0.008	0.007	0	0	0	0	0	0	0.004	0	0	0	0	0	0	0	0
**uncultured_151**	0	0	0	0	0	0.006	0	0	0	0	0.004	0	0	0	0	0.031	0	0	0	0	0	0	0
**uncultured_152**	0.006	0	0	0	0	0.011	0	0.007	0.004	0	0	0	0	0	0	0.007	0	0	0	0	0	0	0
**uncultured_153**	0	0	0	0	0	0.011	0.008	0	0	0.003	0	0	0.025	0	0	0.010	0.026	0	0	0	0	0	0
**uncultured_154**	0	0	0	0	0	0.051	0	0	0	0.003	0	0.424	0	0	0	0	0	0	0	0	0	0	0
**uncultured_155**	0	0	0	0	0	0.023	0	0.004	0.004	0.003	0.004	0.847	0	0.006	0	0	0	0	0	0	0	0	0
**uncultured_156**	0.002	0	0	0	0	0.039	0	0	0	0	0	0	0	0	0.004	0.014	0	0	0	0	0	0	0
**uncultured_157**	0.006	0	0	0.033	0	0.006	0.004	0	0	0	0	0	0	0	0	0	0	0	0	0	0	0	0
**uncultured_158**	0.002	0	0.016	0.037	0	0	0	0	0	0	0	0	0	0	0	0	0	0	0	0	0	0	0
**uncultured_159**	0	0	0	0	0	0.039	0.004	0.004	0.004	0.003	0	0	0	0	0	0.010	0	0	0	0	0	0	0
**uncultured_160**	0.002	0	0	0	0	0.023	0.008	0.011	0	0.007	0	0	0.025	0	0	0	0	0	0	0	0	0	0
**uncultured_161**	0	0	0	0	0.066	0	0	0	0	0	0	0	0	0	0	0	0	0	0	0	0	0	0
**uncultured_162**	0	0	0	0	0	0.017	0.008	0.011	0.004	0.007	0.012	0	0.013	0	0	0	0	0	0	0	0	0	0
**uncultured_163**	0	0	0	0	0	0	0	0	0	0	0	0	0	0	0	0.055	0	0	0	0	0	0	0
**uncultured_164**	0	0	0	0	0	0.006	0	0	0	0	0	0	0	0	0	0.055	0	0	0	0	0	0	0
**uncultured_165**	0	0	0	0	0	0.006	0	0.004	0	0	0	0	0.013	0	0	0.048	0	0	0	0	0	0	0
**uncultured_166**	0.018	0	0	0	0	0.011	0.008	0.004	0	0.003	0	0	0	0	0	0	0	0	0	0	0	0	0
**uncultured_167**	0	0.037	0	0.037	0	0	0	0	0	0	0	0	0	0	0	0	0	0	0	0	0	0	0
**uncultured_168**	0.003	0	0	0	0	0.011	0.004	0	0	0.003	0.008	0.424	0	0	0	0.027	0.013	0	0	0	0	0	0
**uncultured_169**	0	0	0	0	0	0.051	0	0.007	0	0.024	0	0.212	0.013	0.013	0	0	0	0	0	0	0	0	0
**uncultured_170**	0.029	0	0	0	0	0.006	0	0	0.004	0.007	0	0	0	0	0	0	0	0	0	0	0	0	0
**uncultured_171**	0.014	0.010	0	0	0	0.011	0.008	0.004	0.009	0.003	0.008	0.212	0	0	0	0	0	0	0	0	0	0	0
**uncultured_172**	0.006	0	0	0	0	0.051	0.012	0.007	0.013	0.020	0	0.424	0.013	0	0	0	0	0	0	0	0	0	0
**uncultured_173**	0	0	0	0.037	0	0.034	0.004	0.004	0.009	0.017	0.012	0.424	0.025	0.006	0	0	0	0	0	0	0	0	0
**uncultured_174**	0	0	0	0	0	0.045	0.012	0.011	0.022	0.030	0	0.212	0.038	0.032	0	0	0	0	0	0	0	0	0

Continuation of Tab. S2

Sampling site	Nueces River mouth					Gulf of Mexico										Palmyra Atoll	Nankai Trough		South Atlantic	Equatorial Pacific		North Pond	
Depth	0 - 2	2 - 4	4 - 6 (cmbsf)	6 - 8	8 - 10	0 - 2	2 - 4	4 - 6	6 - 8	8 - 10 (cmbsf)	10 - 12	12 - 14	14 - 16	16 - 18	18 - 20	0 - 2 cmbsf	0.2 - 10 (mbsf)	10 - 20	0.2 - 10 mbsf	0.2 - 10 (mbsf)	20 - 100	0.2 - 10 (mbsf)	20 - 100
**uncultured_175**	0	0	0	0	0.183	0	0	0	0	0	0	0	0	0	0	0	0	0	0	0	0	0	0
**uncultured_176**	0.008	0	0	0.093	0	0.039	0	0.004	0	0.017	0.004	0	0.025	0	0	0	0	0	0	0	0	0	0
**uncultured_177**	0	0.161	0	0	0	0	0	0	0	0	0	0	0	0	0	0	0	0	0	0	0	0	0
**uncultured_178**	0	0	0	0	0.235	0	0	0	0	0	0	0	0	0	0	0	0	0	0	0	0	0	0
**uncultured_179**	0.005	0	0	0	0	0.118	0.021	0.004	0.027	0.034	0.008	0.636	0.013	0.013	0	0.007	0	0	0	0	0	0	0
**uncultured_180**	0.032	0	0.004	0.037	0	0.101	0	0.004	0.013	0.007	0.004	0.212	0	0.006	0	0.041	0	0	0	0	0	0	0
**uncultured_181**	0	0	0	0	0	0	0	0	0	0	0.0041	0	0	0	0	0.215	0.128	0	0	0	0	0	0
**uncultured_182**	0	0	0	0	0	0.163	0.029	0.045	0.062	0.034	0.012	0.847	0.126	0.013	0	0	0	0	0	0	0	0	0
**uncultured_183**	0	0	0	0	0.451	0	0	0	0	0	0	0	0	0	0	0	0	0	0	0	0	0	0
**uncultured_184**	0.012	0.060	0	0.056	0	0.113	0.029	0.019	0.027	0.027	0.016	1.695	0.075	0	0.007	0	0	0	0	0	0	0	0
**uncultured_185**	0	0	0	0	0	0	0	0	0	0	0	0	0	0	0	0.003	0	0	0	0	0	0	0
**uncultured_186**	0	0	0	0	0	0	0	0	0	0	0	0	0	0	0	0.010	0	0	0	0	0	0	0
**uncultured_187**	0	0	0	0	0	0.310	0.004	0	0	0.003	0	0	0	0	0	0.017	0	0	0	0	0	0	0
**uncultured_188**	0	0	0	0	0	0	0	0	0	0	0	0	0	0	0	0.017	0	0	0	0	0	0	0
**uncultured_189**	0	0	0.008	0	0	0	0	0	0	0	0	0	0	0	0	0	0	0	0	0	0	0	0
**uncultured_190**	0	0	0	0	0	0.017	0	0	0	0	0	0	0	0	0	0	0	0	0	0	0	0	0
**uncultured_191**	0.002	0	0	0	0	0.028	0	0	0	0	0	0	0	0	0	0	0	0	0	0	0	0	0
**uncultured_192**	0	0	0	0	0	0.034	0.008	0.004	0.004	0.003	0.004	0	0	0	0	0.003	0	0	0	0	0	0	0
**uncultured_193**	0	0	0	0	0	0.039	0.008	0.004	0.013	0	0	0	0.013	0	0	0.003	0	0	0	0	0	0	0
**uncultured_194**	0	0	0	0	0	0	0.004	0	0	0	0.004	0	0	0	0	0	0	0	0	0	0	0	0
**uncultured_195**	0	0	0	0	0	0.006	0	0	0	0	0	0	0	0	0	0	0	0	0	0.004	0	0	0
**uncultured_196**	0	0	0	0	0	0.023	0.004	0	0	0	0	0.212	0	0	0	0	0	0	0	0	0	0	0
**uncultured_197**	0	0	0.008	0	0	0	0	0	0	0	0	0.424	0	0.006	0	0	0.026	0	0	0	0	0	0
**uncultured_198**	0	0	0	0	0	0.011	0	0.011	0	0	0	0.424	0.013	0	0	0	0	0	0	0	0	0	0
Wenxinia	0	0	0	0	0	0.034	0	0	0	0	0	0	0	0	0	0.051	0	0	0	0	0	0	0
