# Supplementary material for: The Majority of Active Rhodobacteraceae in Marine Sediments Belong to Uncultured Genera: A Molecular Approach to Link Their Distribution to Environmental Conditions
Source: Front Microbiol. 2019 Apr 2;10:659. doi: 10.3389/fmicb.2019.00659 (PMC6454203; doi:10.3389/fmicb.2019.00659)
Supplement: Supplementary file 3 [file Data_Sheet_3.PDF]

Tab. S3 Phylogenetic classification of genera within the different subgroups of the *Rhodobacteraceae.*

***Rhodobacter* group**	***Rhodovulum* group**	***Amaricoccus* group**	***Paracoccus* group**	***Stappia* group**	***Roseobacter* group**		
25 genera	3 genera	8 genera	3 genera	11 genera	126 genera		
Albirhodobacter	Albidovulum	Albimonas	Methylarcula	Acuticoccus	Actibacterium	Marimonas	Pseudoroseicyclus
Cereibacter	Jhaorihella	Amaricoccus	Paracoccus (=Thiosphaera)	Agaricicola	Aestuariibius	Marinibacterium	`Pseudoroseobacter´ *
Confluentimicrobium	Rhodovulum	Halovulum	Piezobacter	Ahrensia	Aestuariicoccus	`Marinosulfonomonas´	Pseudoroseovarius
Defluviimonas		Limibaculum		Labrenzia	Aestuariihabitans	Marinovum	Pseudoruegeria
Falsirhodobacter		Oceanicella		Nesiotobacter	Aestuariivita	Maritimibacter	Pseudoseohaeicola
Frigidibacter		Pleomorphobacterium		Pannonibacter	Aliiroseovarius	Marivita (=Gaetbulicola)	Psychromarinibacter*
Gemmobacter (=Catellibacterium)		Rubribacterium		Polymorphum	Aliisedimentitalea	Marivivens	Puniceibacterium
Haematobacter		Rubrimonas		Pseudovibrio	Alkalimicrobium	Monaibacterium	Rhodosalinus
Halodurantibacterium				Rhodothalassium	Amylibacter	Nautella	Roseibacterium
`Natronohydrobacter´				Roseibium	`Antarcticicola´*	Neptunicoccus	Roseicyclus
Paenirhodobacter				Stappia	Antarctobacter	Nereida	Roseisalinus
Pararhodobacter					Aquicoccus	Nioella	Roseivivax
Plastorhodobacter					Aquimixticola	Oceanibulbus	Roseobacter
Pseudorhodobacter					Ascidiaceihabitans	Oceanicola	Roseovarius
Rhodobaca					Boseongicola	Oceaniglobus	Rubellimicrobium
Rhodobacter					Brevirhabdus	Oceaniovalibus	Rubricella
Rhodobaculum					Celeribacter	`Oceanobacterium´*	Ruegeria (=Silicibacter)
Roseibaca					Citreicella	Octadecabacter	Sagittula
Roseibacula					Citreimonas	Pacificibacter	Salinihabitans
Roseicitreum					Cribrihabitans	Palleronia	Salinovum
Roseinatronobacter					Dinoroseobacter	Paradonghicola	Salipiger
Sinorhodobacter					Donghicola	Paraphaeobacter	Sedimentitalea
Tabrizicola					Epibacterium	Pelagibaca	Sediminimonas
`Tetracoccus´					Halocynthiibacter	Pelagicola	Seohaeicola
Thioclava					Hasllibacter	Pelagimonas	Shimia
					Huaishua	Phaeobacter	Silicimonas
					Hwanghaeicola	Planktomarina	Sulfitobacter (=Staleya)
					Jannaschia	Planktotalea	Tateyamaria
					Jindonia	Pontibaca	Thalassobacter
					Ketogulonicigenium	Ponticoccus	`Thalassobium´*
					Lacimonas	Pontivivens	Thalassobius
					Leisingera	Poseidonocella	Thalassococcus
					Lentibacter	Primorskyibacter	Thiobacimonas
					Litoreibacter	Profundibacterium	Tranquillimonas
					Litorimicrobium	Pseudodonghicola	Tritonibacter
					Litorisediminicola	Pseudohalocynthiibacter	Tropicibacter (=Phaeomarinomonas)
					Litorisediminivivens	Pseudomaribius	Tropicimonas
					Loktanella	`Pseudomarivita´*	Vadicella
					Lutimaribacter	Pseudooceanicola	Wenxinia
					Maliponia	Pseudooctadecabacter	Xuhuaishuia
					Mameliella	Pseudopelagicola	Yangia
					Maribius	Pseudophaeobacter	Youngimonas

`genus not validly published´, *classification in subgroup not clear
